# Functional insight into cyclin-dependent kinase (CDK)7 via chemical inhibition of the priority fungal pathogen *Cryptococcus neoformans*

**DOI:** 10.1128/mbio.02898-25

**Published:** 2025-10-31

**Authors:** Pooja Sethiya, Desmarini Desmarini, Bethany Grace Bowring, Kyle Cesar, Michael J. Boucher, Angela L. Wei, Hiten D. Madhani, Ben Crossett, Angela Connelly, Catriona L. Halliday, Sharon C.-A. Chen, Joey Lai, Koon Ho Wong, Julianne Teresa Djordjevic

**Affiliations:** 1 Centre for Infectious Diseases and Microbiology, Westmead Institute for Medical Research 107640, Westmead, New South Wales, Australia; 2 Faculty of Medicine and Health, Sydney Institute for Infectious Diseases, University of Sydney, Camperdown, New South Wales, Australia; 3 Department of Biochemistry and Biophysics, University of California 117253 , San Francisco, California, USA; 4 Sydney Mass Spectrometry, University of Sydney, Camperdown, New South Wales, Australia; 5 Centre for Infectious Diseases and Microbiology Laboratory Services, Institute for Clinical Pathology and Medical Research, New South Wales Health Pathology, Westmead Hospital, Westmead, New South Wales, Australia; 6 Westmead Hospital 8539, Westmead, New South Wales, Australia; 7 Scientific Platforms, Westmead Institute for Medical Research, Westmead, New South Wales, Australia; 8 Faculty of Health Sciences, University of Macau, Macau SAR, China; Yonsei University, Seoul, Republic of Korea

**Keywords:** CDK7, CAK complex, *Cryptococcus neoformans*, cell cycle, RNAPII, transcription, splicing, MAPK, Hog1, mevociclib, SY-1365, SY-5609, CDK7-IN-3, samuraciclib

## Abstract

Cyclin-dependent kinase (CDK)7 regulates the cell cycle and transcription in higher eukaryotes, but its function and the antifungal potential of CDK7 inhibitors remain unexplored in fungal pathogens. CDK7 inhibitors were identified to probe CDK7 function in the priority fungal pathogen, *Cryptococcus neoformans* (*Cn*). The CDK7 ortholog in *Cn* was tagged with mNeonGreen and shown to form a nuclear-localized, CDK-activating kinase (CAK) complex with cyclin H and assembly factor Mat1. The pulled-down CAK complex was demonstrated to be active and was used to identify several human CDK7 inhibitors that also inhibit *Cn*CDK7 enzyme activity. In addition to inhibiting *Cn*CDK7 enzyme activity, the compound mevociclib (SY-1365) most significantly inhibited fungal growth and was deployed to probe *Cn*CDK7 function using multi-omics analysis, flow cytometry, and Western blotting, with roles in transcription via RNA polymerase II phosphorylation, mRNA splicing, and progression through G_2_/M of the cell cycle identified. Inhibition of CDK7 also suppressed MAP kinase (Hog1) signaling. Notably, compound SY-1365 and related inhibitors synergized with licensed, membrane-targeting antifungals at low micromolar concentrations against both *C. neoformans* and *Cryptococcus gattii*. Our findings establish SY-1365 as a powerful chemical probe to define CDK7 function in *Cn* and suggest that its role more closely resembles that in human cells than in model fungi. Furthermore, given that CDK7 inhibitors are used in oncology, our findings identify CDK7 inhibitors as promising antifungal candidates for combination therapy with licensed antifungals, with the potential to overcome current challenges associated with toxicity and antifungal resistance.

**IMPORTANCE:**

*Cn*, a basidiomycete causing meningoencephalitis with 40%–60% mortality, was recently assigned as a “critical” priority pathogen by the World Health Organization. *Cn* commonly affects AIDS patients, organ transplant recipients, and individuals with hematological malignancies, yet current treatments are limited by toxicity or resistance due to prolonged therapy. Given that CDK7 inhibitors kill leukemia cells, and Cn proliferation in host tissues mimics tumor-like growth, we investigated whether their utility as anticancer agents extends to antifungal activity and elucidation of CDK7 function in *Cn*. Use of SY-1365, which was both antifungal and CnCDK7 inhibitory, identified roles for CnCDK7 in all stages of transcription, mRNA splicing, and cell cycle regulation. The antifungal activity of SY-1365 was also markedly enhanced in combination with membrane-targeting antifungals. Together, our findings highlight CDK7 inhibitors as valuable tools to study CDK7 function in Cn and as potentially promising antifungals in combination with licensed antifungals.

## INTRODUCTION

In higher eukaryotes, cyclin-dependent kinases (CDKs) regulate cell cycle progression and transcription and are activated by phosphorylation of the threonine (T) residue within their CDK activation T-loop (1–3). The CDK responsible for this activation is CDK7 (4). CDK7 associates with cyclin H (CycH) and the ring finger assembly factor, Mat1, to form a CDK-activating kinase (CAK) complex (5, 6) where CDK7 phosphorylates CDK1, CDK2, CDK4, and CDK6 to promote the various stages in the cell cycle. The CAK complex can also associate with the general transcription factor, TFIIH, to initiate transcription. The CAK–TFIIH association regulates transcription, with CDK7 phosphorylating Ser5 in the C-terminal domain (CTD) of the RNA polymerase II (RNAPII) subunit Rpb1 within the pre-initiation complex (PIC). This phosphorylation event initiates transcription by promoting the release of RNAPII from PIC (7, 8). CDK7 also phosphorylates CDK9 (9). CDK9 then phosphorylates Ser2 in the Rpb1 CTD, which is important for several transcriptional processes, including elongation and splicing factor recruitment (8, 10). Because CAK function is essential, elucidation of CAK function has relied predominantly on the use of analog-sensitive mutants and CDK7 inhibitors (11, 12). Given that CDK7 is upregulated in cancer cells, CDK7 inhibitors are emerging as a promising chemotherapeutic option to minimize side effects, with some inhibitors having entered clinical trials to treat a variety of cancers (11, 13, 14). Unlike normal cells, tumor cells depend on CDK7, providing a rationale for investigating CDK7 inhibitors as chemotherapeutic agents.

CDK7 function is also essential in model non-pathogenic yeast. However, its function has diverged from that of higher eukaryotes. In the budding yeast, *Saccharomyces cerevisiae,* the CDK7 homolog Kin28 forms a trimeric complex with a cyclin (Ccl1) and a transcription factor (Tfb3). In contrast to higher eukaryotes, Kin28-Ccl1-Tfb3 does not function as a CAK complex to phosphorylate Cdc28/CDK1 and promote cell cycle progression (15). However, when associated with TFIIH, Kin28-Ccl1-Tfb3 is involved in transcriptional and co-transcriptional regulation, with Kin28 phosphorylating Ser2 and Ser5 on the CTD of Rpb1 (15–18). In the fission yeast, *Schizosaccharomyces pombe*, the CDK7 homolog, Mcs6, forms a CAK complex with Mcs2 and Pmh1. Mcs6-Mcs2-Pmh1 has an essential regulatory role in transcription by phosphorylating Rpb1 to control gene expression and a redundant role in cell cycle regulation (16, 17).

CDK7 function has not yet been studied in fungal pathogens, including in the priority fungal pathogen *Cryptococcus neoformans* (*Cn*) (19). CDK7, cyclin H, and Mat1 homologs are present in the *Cn* genome (20), and *Cn*CDK7 function is likely to be essential based on a saturation transposon mutagenesis study (21) and CAK knockout strains not being represented in a *Cn* kinome library (22) or a recently produced *Cn* deletion strain collection (23). We hypothesized that inhibitors of human CDK7 could also potentially inhibit CDK7 from *Cn* and thus provide tools to elucidate its function. Because CDK7 function is not amenable to study using gene deletion approaches, we introduced molecular tags to the CDK7, cyclin H, and Mat1 homologs in *Cn* to investigate their association and cellular localization. The tagged enzyme was also used to develop an enzyme assay to identify a CDK7 inhibitor that both inhibits *Cn*CDK7 enzyme activity *in vitro* and is significantly bioavailable to reduce fungal growth at low micromolar concentrations as a stand-alone agent. We then used this inhibitor as a tool in phosphoproteomics and RNA-seq studies, as well as in flow cytometry and Western blotting experiments, to investigate CDK7 function in *Cn*.

## RESULTS

### *Cn*CDK7 associates with cyclin H and Mat1 to form a nuclear-localized CAK complex

**Fig 1:**
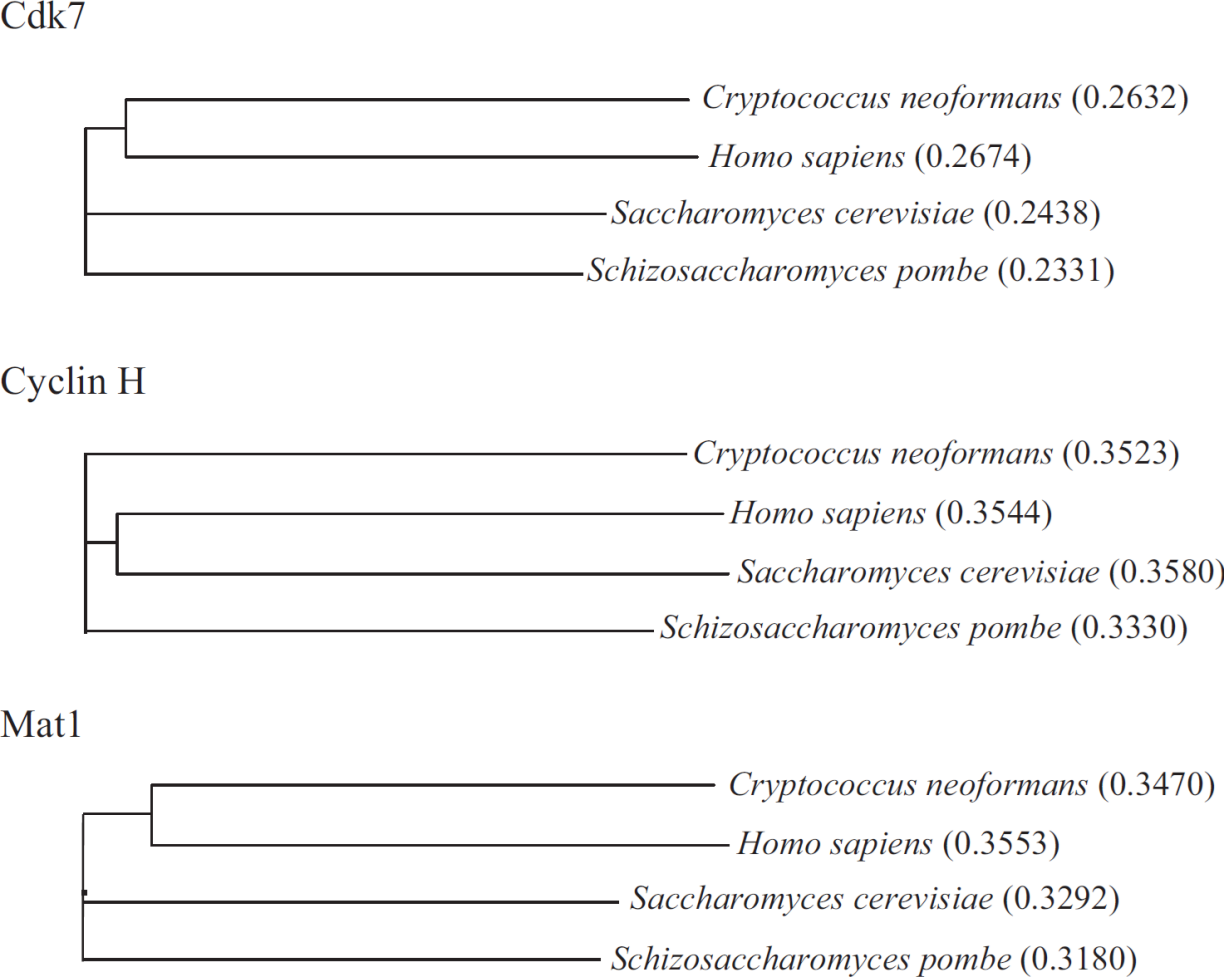
Phylogenetic/guide trees obtained following multiple amino acid sequence alignment of the CDK7, cyclin H, and Mat1 homologs from *C. neoformans* (*Cn*), *Homo sapiens* (*Hs*), *S. cerevisiae* (*Sc*), and *S. pombe* (*Sp*). The trees show that *Cn*CDK7 and *Cn*Mat1 proteins cluster with the human homologs. *Cn*CDK7 (CNAG_06445 and XP_012053336.1), *Hs*CDK7 (NP_001790.1), *Sc*CDK7/*Sc*Kin28 (NP_010175.1), *Sp*CDK7/*Sp*Mcs6 (NP_596349.1), *Cn*Cyclin H (CNAG_04405 and XP_012051791.1), *Hs*CycH (NP_001230.1), *Sc*CycH/*Sc*Ccl1 (NP_015350.1), *Sp*CycH/*Sp*Mcs2 (NP_595776.1), *Cn*Mat1 (CNAG_05877 and XP_012050601.1), *Hs*Mat1 (NP_002422.1), *Sc*Mat1/*Sc*Tfb3 (NP_010748.3), and *Sp*Mat1/*Sp*Pmh1 (NP_596334.1). Numbers represent sequence distances.

A homology search of the *C. neoformans* genome (20) identified homologs of *CDK7* (CNAG_06445), *MAT1* (CNAG_05877), and CycH (CNAG_04405). Sequence alignment and phylogenetic analysis (24) of the human and fungal CDK7 amino acid sequences revealed that *Cn*CDK7 is more similar to human CDK7 than are the CDK7 homologs from the ascomycetes *S. cerevisiae* (Kin28) and *S. pombe* (Mcs6). Accordingly, human CDK7 and *Cn*CDK7 cluster together in the phylogenetic tree (Fig. 1). *Cn*Mat1, but not *Cn*CycH, also clusters with the human homolog (Fig. 1).

**Fig 2:**
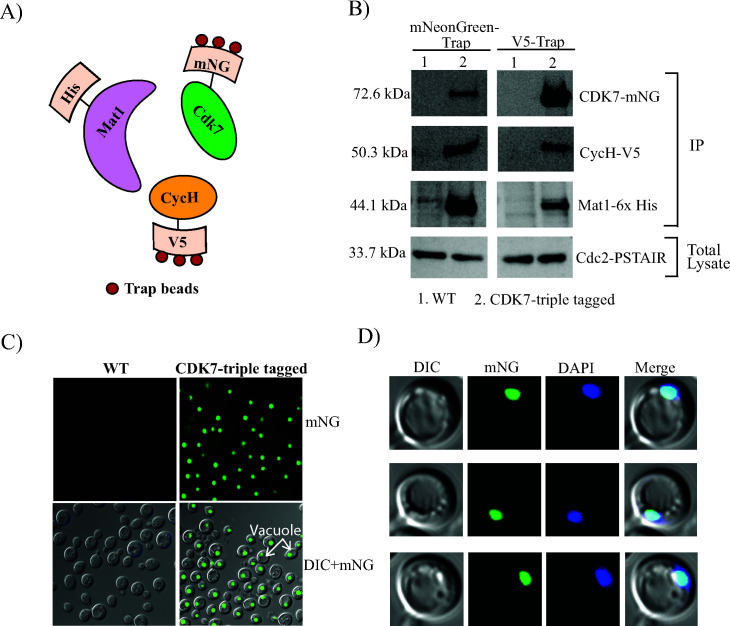
*Cn*CDK7 forms a CAK complex with Mat1 and cyclin H that localizes to the nucleus. (**A**) Schematic depicting the addition of the mNeonGreen (mNG), V5 epitope (V5), and 6× His (His) tags on CDK7, CycH, and Mat1, respectively, in the CDK7 triple-tagged strain as described in the Supplemental Method. (**B**) Immunoprecipitation (IP) and Western blotting with antibodies to each tag demonstrates formation of a CAK complex: in the left panel, *Cn*CDK7 was immunoprecipitated from the CDK7 triple-tagged strain with anti-mNG trap and subjected to SDS-PAGE and Western blotting with anti-mNG, anti-6× His, and anti-V5, detecting CDK7, CycH, and Mat1, respectively (lane 2). In the right panel, CycH was immunoprecipitated from the CDK7 triple-tagged strain with V5-Trap, followed by Western blotting with the various antitag antibodies (lane 2). In both panels, the non-tagged parent KN99 WT strain (WT) was taken through the same procedure as a negative control (lane 1). The total lysates used for each IP were probed with anti-PSTAIR to demonstrate the presence of Cdc2 in all IP samples. Epifluorescence microscopy validated the fluorescence of *Cn*CDK7 in the CDK7 triple-tagged strain (**C**) and the nuclear localization of the *Cn*CAK complex (**D**). DIC, Differential interference contrast.

To determine if CDK7 forms a complex with cyclin H (CycH) and MAT1 in *Cn*, strain CM2448 was created (hereafter referred to as the CDK7 triple-tagged strain), where CDK7, CycH, and MAT1 were tagged with mNeonGreen (mNG), V5 epitope, and 6× histidine, respectively (Fig. 2A). The method describing the sequential tagging of each CAK complex component to create the *Cn*CDK7 triple-tagged strain is described in Fig. 2A and in the Supplemental Method and Table S1 through S3 (25). The *Cn*CDK7 triple-tagged strain has similar growth and virulence characteristics (Fig. S1) as wild-type (WT) *Cn*, confirming that the addition of the tags does not impact cellular physiology. CDK7, CycH, and Mat1 association in *Cn* was investigated by performing pull-down experiments with the CDK7 triple-tagged strain and mNG-Trap (Fig. 2B, left-hand panel) and V5-Trap (Fig. 2B, right-hand panel) followed by Western blotting with anti-mNG, anti-V5, and anti-6× His antibodies. In both pull-downs, bands with the expected molecular weight of CDK7, CycH, and Mat1 were detected in the CDK7 triple-tagged strain but not in the untagged KN99 strain, confirming that all three components associate to form a CAK complex in *Cn*. To determine the location of the CAK complex in *Cn*, mNG-CDK7 was visualized by fluorescence microscopy as bright punctate structures (Fig. 2C) that coincided with 4′,6-diamidino-2-phenylindole (DAPI)-stained nuclei (Fig. 2D). No fluorescence was detected in the KN99 control strain.

### CDK7 inhibitors with chemotherapeutic efficacy also inhibit *Cn*CDK7

**Fig 3:**
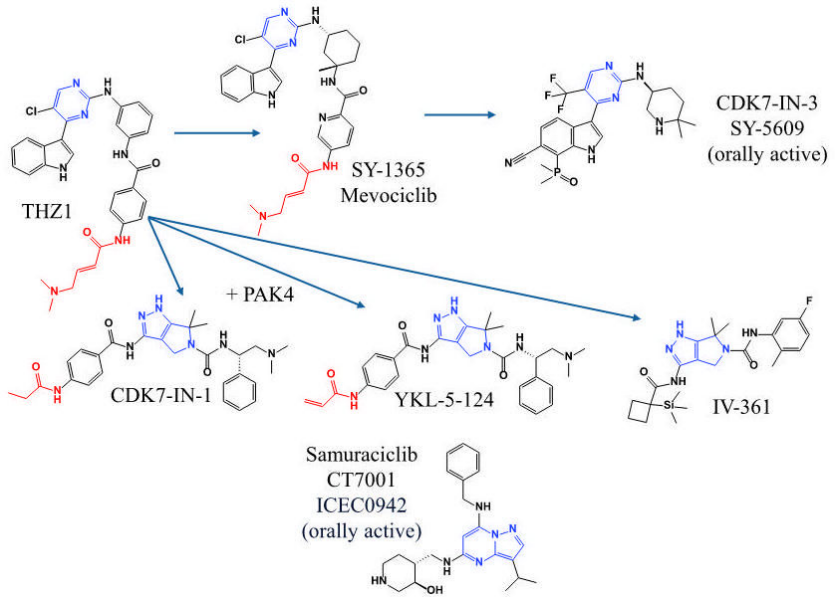
Chemical structures of the human CDK7 inhibitors tested in this study. Inhibitors are arranged based on their molecular scaffold (in blue): pyrimidine (THZ1, SY-1365, and CDK7-IN-3), pyrrolidinopyrazole from p21-activated kinase (PAK4) (CDK7-IN-1, YKL-5-124, and IV-361) and pyrazolopyrimidine (samuraciclib). Red regions indicate the covalent warhead. THZ1 served as a molecular starting point for the creation of all compounds (indicated by the arrows), except Samuraciclib.

Since human and *Cn*CDK7 cluster together in a phylogenetic/guide tree analysis (Fig. 1), we hypothesized that human CDK7 inhibitors, which are under investigation as chemotherapeutic agents, also inhibit *Cn*CDK7, providing tools to study the antifungal potential of inhibiting *Cn*CDK7 and CDK7 function. We selected a range of inhibitors that are potent and selective for human CDK7 and which represented structural diversity across three distinct molecular classes (Fig. 3). THZ1, the parent compound from which two of the molecular classes were derived, was included in the study. Although THZ1 strongly inhibits CDK7, it also targets other CDKs and is therefore less selective (11). SY-1365 (26), samuraciclib (13), and CDK7-IN-3 (27, 28) were included because they have advanced to clinical trials or clinical use as chemotherapeutic agents. Samuraciclib and CDK7-IN-3 are also orally available, which is considered the gold standard in drug development. Several inhibitors also had a covalent warhead, which is designed to slow the inhibitors’ off rate and potentially increase efficacy and bioavailability (11).

**Fig 4:**
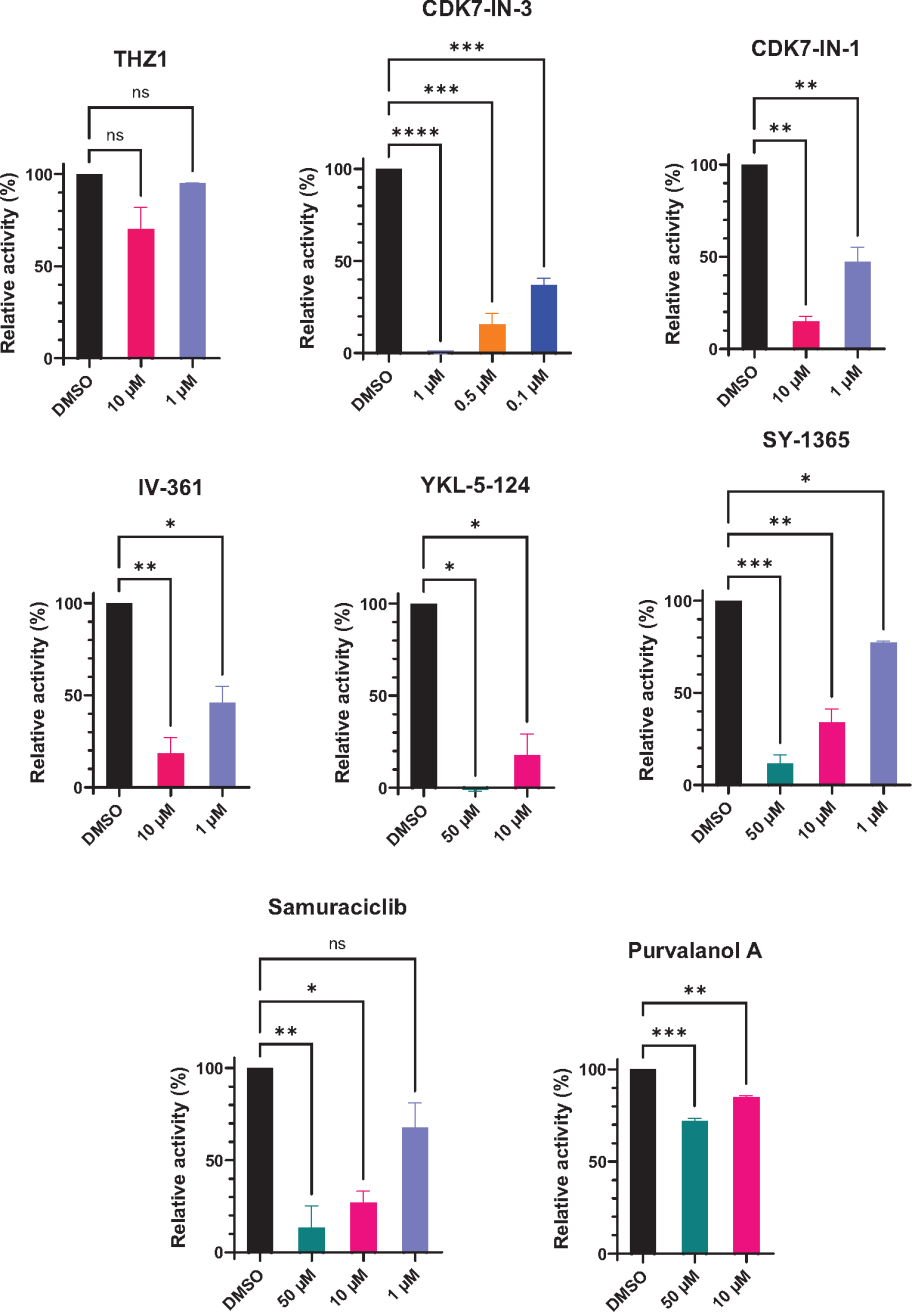
*Cn*CDK7 is inhibited by human CDK7 inhibitors. Kinase assays were performed over a 300 min time course in the absence (DMSO) and presence of the indicated concentrations of each CDK7 inhibitor, using pulled-down CAK, CDK7 peptide substrate, and Kinase-Glo reagent. The latter allowed ATP consumption (due to the phosphorylation of the peptide substrate by CDK7) to be measured as a relative luminescence unit. The full dose–response time curves are shown in Fig. S2. Only CDK7 enzyme activity at 300 min is plotted and is expressed as “relative activity (%)” after normalization as described in Materials and Methods. The CDK1/CDK2/CDK4/CDK5 inhibitor, Purvalanol A, is included as a negative control. The results represent the mean relative activity ± SEM (*n* = 2–3 independent experiments). Statistical analysis was performed using ordinary one-way analysis of variance with Dunnett’s multiple comparison test, comparing each concentration to the DMSO control. *P < 0.05, **P < 0.01, ***P < 0.001, ****P < 0.0001. ns, not significant.

The ability of these inhibitors to inhibit *Cn*CDK7 enzyme activity was investigated using pulled-down *Cn*CAK and a peptide substrate that is phosphorylated by human CDK7. Dose–response curves were conducted over a time course (Fig. S2), and the last point (300 min) was plotted against a no-inhibitor (dimethyl sulfoxide [DMSO]) control (Fig. 4). Except for THZ1, the parent compound (29) used to create many of the more potent human CDK7 inhibitors (Fig. 3), all the inhibitors suppressed the relative activity of *Cn*CDK7 in a dose-dependent manner (Fig. 4 and Fig. S2). SY-5609 (CDK7-IN-3), which is orally active, was the most inhibitory, followed by CDK7-IN-1 and IV-361, and SY-1365 and orally active samuraciclib. Purvalanol A, a CDK1/CDK2/CDK4/CDK5 inhibitor used as a negative control, caused minimal suppression of *Cn*CDK7 activity.

### SY-1365 is the most antifungal CDK7 inhibitor

**Fig 5:**
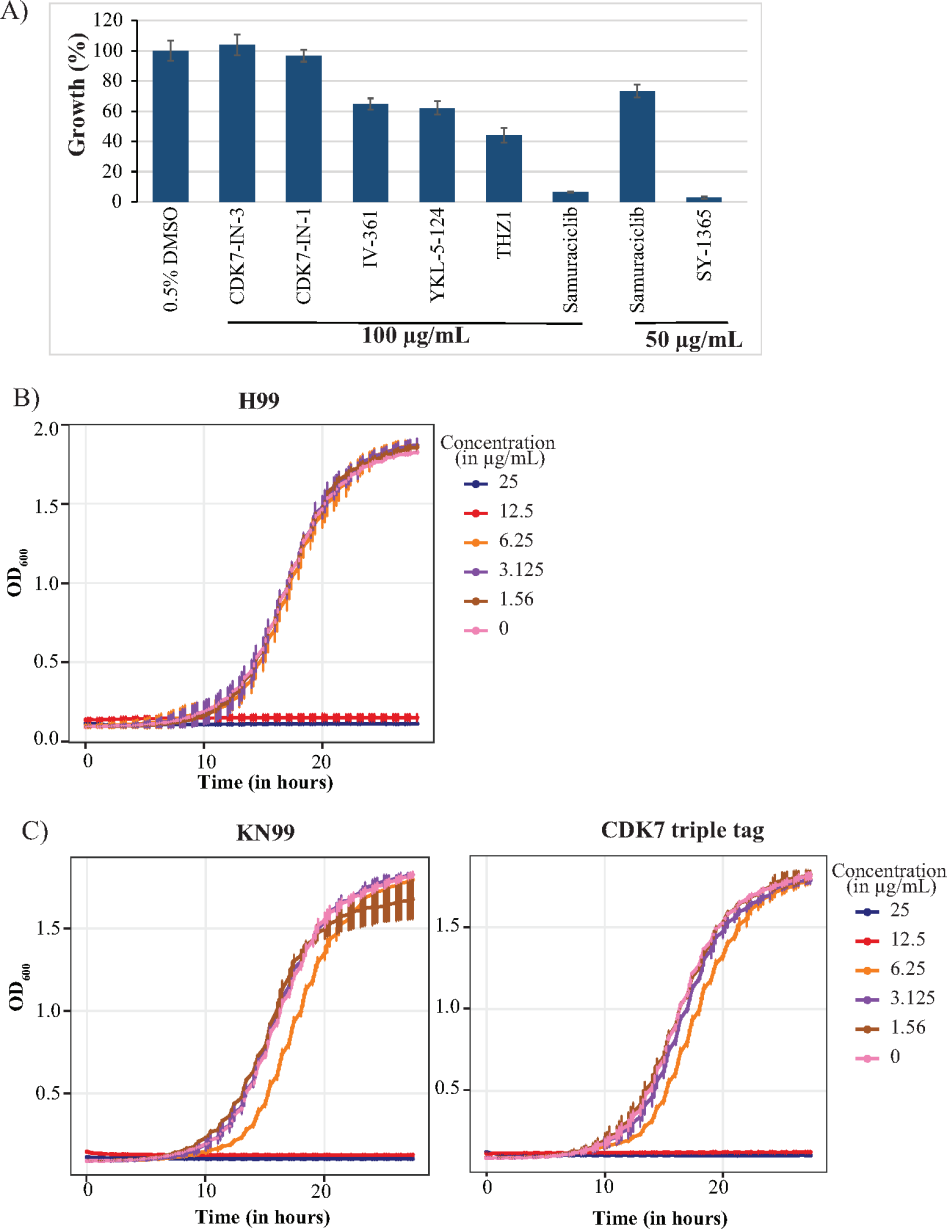
Impact of SY-1365 on *Cn* growth. (**A**) An overnight culture of *Cn* strain H99 was used to seed a fresh culture to OD_600_/mL = 0.01, which was allowed to grow for 17 h in the presence of each drug at the indicated concentration. Growth inhibition was calculated as a percentage (%) relative to the no drug treatment control (0.5% DMSO). Dose–response curves for *Cn* strain H99 (**B**), *Cn* strain KN99 (**C**), and the triple-tagged strain in the KN99 background (**D**) were generated in a 96-well plate in the presence and absence of SY-1365. Growth was determined by recording OD_600_ readings every 20 min using an Agilent Biotek log phase 600 plate reader.

Next, we tested whether these CDK7 inhibitors inhibit growth of the *Cn* H99 WT strain in a broth culture assay. The results demonstrate that inhibitor potency against *Cn*CDK7 did not correlate with antifungal activity (Fig. 5A): while CDK7-IN-3 was the most potent *Cn*CDK7 inhibitor (Fig. 3), it did not inhibit fungal growth, even at the highest soluble concentration (100 µg/mL). THZ1 modestly inhibited growth at 100 µg/mL but was the weakest inhibitor of *Cn*CDK7 enzyme activity. Despite being a weak inhibitor of *Cn*CDK7, the antifungal effect of THZ1 is most likely due to it inhibiting other CDKs. CDK7-IN-1, IV-361, and YKL-5-124 modestly inhibited both fungal growth and *Cn*CDK7 enzyme activity. SY-1365, followed by samuraciclib, was the most antifungal, and both were moderate inhibitors of *Cn*CDK7 enzyme activity. To further investigate the antifungal activity of SY-1365, dose–response experiments were performed over 28 h (Fig. 5B). The results show that SY-1365 concentrations of ≥12.5 µg/mL completely inhibited growth of the H99 strain, consistent with a fungicidal effect. An SY-1365 dose–response curve was also performed on the KN99 and CDK7 triple-tagged strains, and the results showed that they had a drug susceptibility profile similar to the H99 strain, with ≥12.5 µg/mL of SY-1365 completely inhibiting growth (Fig. 5C and D). This confirmed that the addition of the molecular tags does not impact CAK function and that, as expected, SY-1365 has a similar inhibitory effect on the KN99 and H99 strains. Overall, the results demonstrate that SY-1365 has the most antifungal activity and that this antifungal activity correlates with its *Cn*CDK7 enzyme inhibition. SY-1365 is therefore the most suitable inhibitor to study CDK7 function in *Cn*.

### Antifungal activity of CDK7 inhibitors is enhanced by membrane-perturbing antifungals

Next, we investigated whether the lack of a correlation between *Cn*CDK7 enzyme inhibition and antifungal activity could be due to differences in the ability of the inhibitors to penetrate fungal cells. The antifungal activity of SY-1365, CDK7-IN-3, and samuraciclib against *Cn* and *Cryptococcus gattii* (*Cg*) was therefore tested in the presence of membrane-perturbing antifungals—azoles and polyenes—using a standardized checkerboard susceptibility assay. Although SY-1365 and samuraciclib are the most antifungal agents on their own, we wanted to determine whether their activity could be further improved in combination therapy. Samuraciclib is also orally active as a chemotherapeutic agent and hence as a potential antifungal agent. CDK7-IN-3 was chosen because it most potently inhibited *Cn*CDK7 enzyme activity and because it is also orally active as a chemotherapeutic agent and, hence, as a potential antifungal agent. Azoles, such as fluconazole, inhibit ergosterol biosynthesis, whereas polyenes, such as amphotericin B (AmB), bind directly to ergosterol, disrupting fungal membrane integrity and function (30–32).

Compared to the shaking broth cultures (Fig. 5A), the MIC for SY-1365 alone against *Cn* strain H99 was one dilution higher in the susceptibility assay (25 µg/mL, Table 1). Against *Cg* strain R265, the MIC ranged from 12.5 to 25.0 µg/mL. Samuraciclib and CDK7-IN-3 had higher MICs (≥100 µg/mL) when tested alone. However, in combination with AmB, the MIC of all three CDK7 inhibitors was markedly reduced (16-fold for SY-1365, 32- to 128-fold for CDK7-IN-3, and 8- to 128-fold for samuraciclib) (Table 1). Thus, at an AmB concentration ≥2-fold below its stand-alone MIC, only 1.56–6.25 µg/mL of SY-1365 or CDK7-IN-3 and 1.56–25.0 µg/mL of samuraciclib were sufficient to inhibit *Cn* and *Cg* growth. The fractional inhibitory concentration index (FICI) values for the CDK7 inhibitor/AmB combinations ranged between 0.53 and 0.63, reflecting additivity. This compares to FICIs of 0.75 and 0.56 (additive) for the AmB/flucytosine combination against *Cn* and *Cg*, respectively. AmB/flucytosine is standard therapy for initial treatment of cryptococcal infections (33, 34). Hence, combining CDK7 inhibitors with AmB results in a similar or more efficacious effect compared to standard of care therapy. In most cases, the efficacy of the CDK7 inhibitors also improved in the presence of fluconazole (MIC for SY-1365, CDK7-IN-3, and Samuraciclib reduced by 8-fold, 128-fold, and 4- to 128-fold, respectively), although mostly higher concentrations of each inhibitor were required to achieve additivity or synergy.

**TABLE 1:** Antifungal activity of SY-1365, samuraciclib, and CDK7-IN-3 against *Cn* and *C. gattii* laboratory strains alone and in combination with AmB or Flu^*a*^*^,^*^*b*^

Drug(s)	For *Cn* (H99)			For *Cg *(R265)		
	Interaction	FICI	MIC (µg/mL)	Interaction	FICI	MIC (µg/mL)
AmB			0.25–0.5			0.25–0.5
Flu			4.0–8.0			8.0
SY-1365			25.0			12.5–25.0
Samuraciclib			>100.0			>100.0
CDK7-IN-3			>100.0			>100.0
SY-1365 + AmB	Additive	0.56	AmB 0.25, SY 1.56	Additive	0.56	AmB 0.25, SY 1.56
SY-1365 + Flu	Additive	1.0	Flu 2.0, SY 12.5	Additive	0.63	Flu 1.0, SY 12.5
Samuraciclib + AmB	Additive	0.63	AmB 0.25, Sam 25.0	Additive	0.51	AmB 0.5, Sam 1.56
Samuraciclib + Flu	Additive	0.51	Flu 2.0, Sam 1.56	Synergy	0.50	Flu 2.0, Sam 50.0
CDK7-IN-3 + AmB	Additive	0.53	AmB 0.25, IN-3 6.25	Synergy	0.50	AmB 0.5, IN-3 1.56
CDK7-IN-3 + Flu	Indifferent	2.0		Additive	0.51	Flu 4.0, IN-3 1.56

^*a*^ MIC in single and combination therapy (µg/mL); FICI ≤ 0.5 is synergistic; 0.5 < FICI ≤ 1.0 is additive; 1.0 < FICI ≤ 4.0 is indifferent; and FICI > 4.0 is antagonistic. The FICIs for the AmB/flucytosine combination against *Cn* and *Cg* were 0.75 and 0.56 (additive) against *Cn* and *Cg*, respectively (data not shown in table).

^*b*^ AmB, amphotericin B; FICI, fractional inhibitory concentration index; Flu, fluconazole; MIC, minimum inhibitory concentration.

Taken together, the structure–activity relationship study in Fig. 4, combined with the growth assays in the presence and absence of membrane-targeting antifungals (Fig. 5 and Table 1), identifies SY-1365 as a suitable, bioavailable inhibitor for studying CDK7 function in *Cn* and provides a foundation for developing even more potent and/or bioavailable inhibitors of *Cn*CDK7 as stand-alone antifungal agents.

### SY-1365 treatment impacts the *Cn* phosphoproteome

As mentioned, *Cn*CDK7 is most likely an essential protein whose function has not been studied. To characterize *Cn*CDK7 function using omics approaches, we used SY-1365, which both inhibits *Cn*CDK7 enzyme activity (Fig. 4) and is the most potent antifungal inhibitor when used alone (Fig. 5). This allowed us to assess the consequences of inhibiting CDK7 on *Cn* cellular function. In contrast, while samuraciclib is also antifungal, it requires much higher concentrations to be effective and is therefore limited by solubility and bioavailability. Use of chemical inhibition to study protein function has advantages over genetic approaches as it does not lead to long-term adaptive changes.

**Fig 6:**
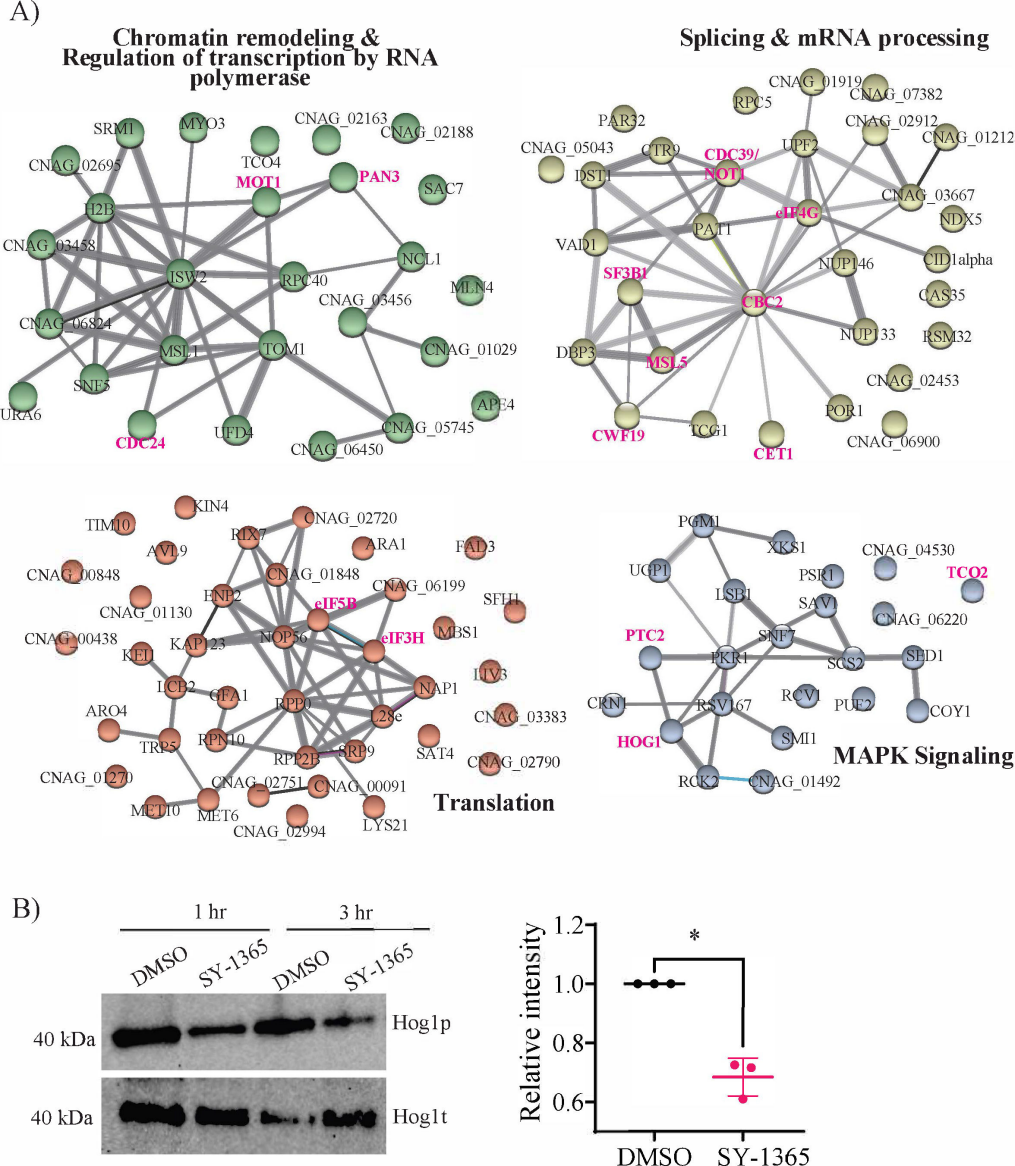
Phosphoproteomic analysis of SY-1356-treated *Cn* strain H99. (**A**) K-means clustering using the STRING database was performed on the 122 proteins (Table S4) that had reduced phosphorylation following CDK7 inhibition with SY-1365 (see the Supplemental Method and Fig. S4 for details). This resulted in four functional clusters (PPI enrichment *P* value <5 × 10⁻⁴). Proteins in pink are discussed in the main text. (**B**) Representative Western blot of *n* = 3 independent experiments (see scatter dot plot), validating reduced phosphorylation of the MAPK, Hog1 (Hog1p) following treatment with SY-1365, as compared to total Hog1 (Hog1t). In the scatter dot plot, the reduction in Hog1p levels after 1 h treatment with SY-1365 is statistically significant (*P = 0.013, unpaired *t*-test; bars represent mean intensity ± SD).

The first omics approach we used to characterize *Cn*CDK7 function was phosphoproteomics as described in the Supplemental Method and Fig. S3. Drug treatment led to a decrease in the phosphorylation of 122 proteins (Table S4), which represent potential direct and indirect substrates of *Cn*CDK7. Search tool for the retrieval of interacting genes/proteins (STRING) network analysis on this *Cn*CDK7 phosphoproteome identified four key functions regulated by *Cn*CDK7: chromatin remodeling/RNA polymerase-mediated transcriptional regulation (1–7); splicing and capping, which are essential second-tier regulators of gene expression; translation; and mitogen-associated kinase (MAPK) signaling (Fig. 6A). Key proteins in these clusters are highlighted in pink and discussed below. Reduced phosphorylation of the putative cell cycle regulator Cdc24 (35) and the transcriptional regulator Mot1 (36) implicates *Cn*CDK7 in cell cycle-associated actin polymerization and transcription initiation, respectively. Reduced phosphorylation of the RNA 5′-triphosphatase Cet1 (37) and the Cbc2 (38) subunit of cap binding protein complex (39), which interacts with Mot1 (36), implicates *Cn*CDK7 in regulating mRNA capping and formation of the PIC. Reduced phosphorylation of Sf3b1 (40), Msl5 (41), and Cwf19 (42) implicates *Cn*CDK7 in spliceosome assembly and regulation. Reduced phosphorylation of the mRNA deadenylation proteins Pan3 and Not1 (Cdc39) and of eIF4G, eIF5B, and eIF3H implicates *Cn*CDK7 in controlling mRNA stability (43) and translation (44), respectively.

Specific proteins with reduced phosphorylation in the MAPK signaling cluster include three components of the hyperosmolar growth (Hog)1 pathway: the MAPK Hog1; the phosphatase Ptc2, which regulates Hog1 entry into the nucleus; and the plasma membrane-associated *t*wo-*co*mponent-like (Tco) stress-sensing kinase, Tco2 (Fig. 6A, in pink). *Cn* uses the Hog1 MAPK pathway to respond to a variety of stresses, including exposure to agricultural fungicides, and to regulate sexual reproduction, virulence, and cross talk with the cAMP signaling cascade (45, 46). Hog1 is constitutively phosphorylated in the *Cn* H99 strain under normal (non-stress) conditions and is rapidly dephosphorylated after exposure to numerous stresses detected by Tco1 and Tco2 (45, 46). Our anti-Hog1 Western blotting results confirm that Hog1 is phosphorylated in untreated *Cn* and that, relative to total Hog1, Hog1 phosphorylation is reduced by SY-1365 treatment (Fig. 6B). This both validates the phosphoproteomic analysis and provides the first evidence of a link between CDK7 function and Hog1 signaling.

### *Cn*CDK7 phosphorylates the CTD of RNAPII

**Fig 7:**
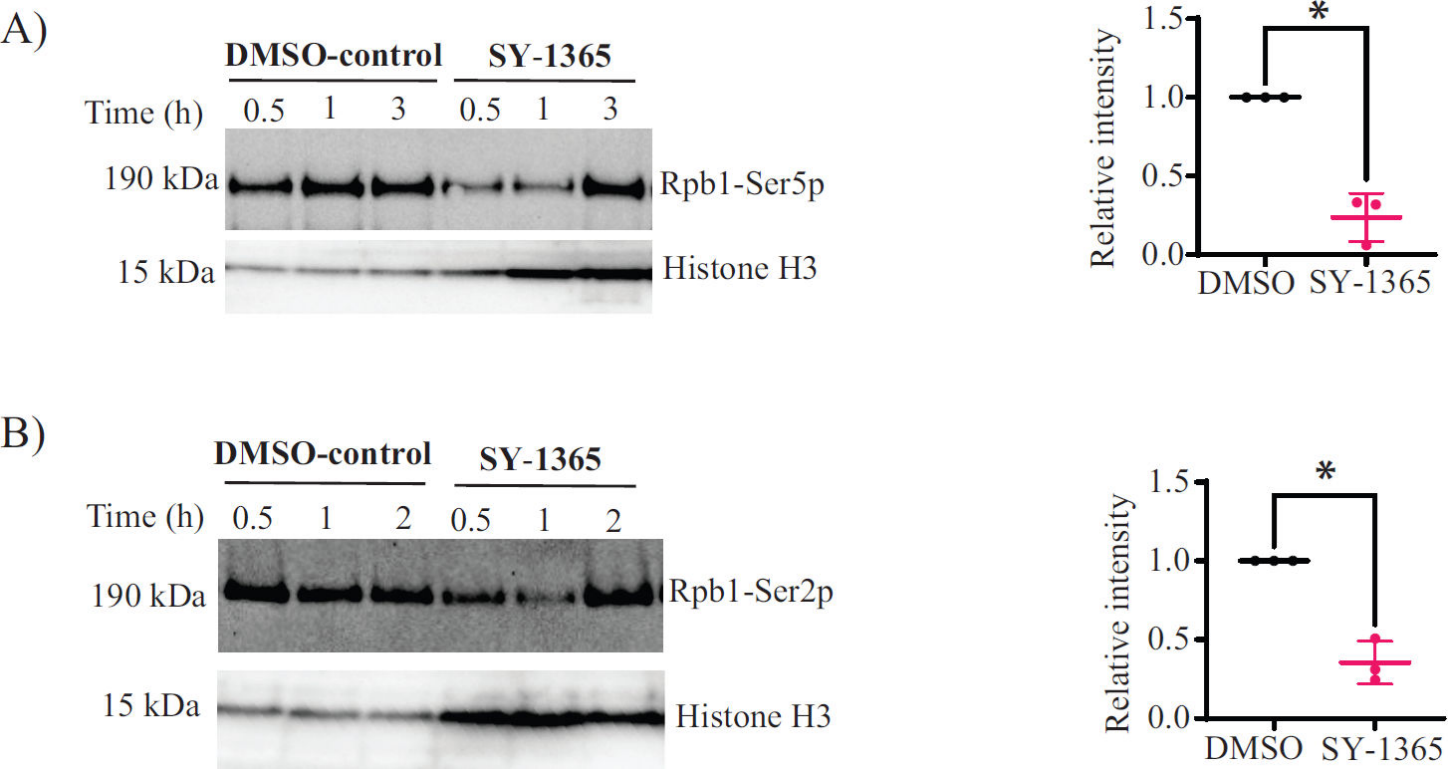
*Cn*CDK7 phosphorylates Ser5 and Ser2 in the CTD of the Rpb1 subunit of RNAPII *in vivo* (**A**) Representative Western blot of three independent experiments (see adjacent scatter dot plot) showing that SY-1365 inhibits phosphorylation of Rpb1 on Ser5 (Rpb1-Ser5p). In the adjacent scatter dot plot, the reduction in Rpb1-Ser5p after 1 h treatment with SY-1365 when normalized to DMSO treatment is statistically significant (*P = 0.013, unpaired *t*-test; bars indicate mean intensity ± SD). (**B**) Representative Western blot of three independent experiments (see adjacent scatter dot plot) showing that SY-1365 inhibits phosphorylation of Rpb1 on Ser2 (Rpb1-Ser2p). In the adjacent scatter dot plot, the reduction in Rpb1-Ser2p after 1 h treatment with SY-1365 when normalized to DMSO treatment is statistically significant (*P = 0.0145, unpaired *t*-test; bars indicate mean intensity ± SD). In panels **A** and **B**, the levels of Rpb1-Ser5p and Rpb1-Ser2p in the SY-1365- and DMSO-treated samples were normalized to histone H3, which was used as a loading control.

In humans and model yeast, TFIIH-associated CDK7 regulates transcription by phosphorylating Ser5 in the Y_1_S_2_P_3_T_4_S_5_P_6_S_7_ heptad sequence of Rpb1, triggering RNAPII escape from the promoter (7, 8). TFIIH-associated CDK7 also phosphorylates CDK9, which in turn phosphorylates Ser2, promoting transcription elongation (9). Given that our phosphoproteomics data implicate *Cn*CDK7 in transcriptional regulation, TFIIH and CDK9 homologs are present in *Cn*, and Y_1_S_2_P_3_T_4_S_5_P_6_S_7_ is phosphorylated by *Cn*CDK7 in the kinase assay (Fig. 4), we investigated whether inhibiting *Cn*CDK7 with SY-1365 impacts phosphorylation of Ser5 and Ser2 *in vivo* using anti-Rpb1-Ser5p and anti-Rpb1-Ser2p Western blotting. In *Cn*, the heptad repeats in the CTD of the Rpb1 subunit of RNAPII contain only six amino acids (Y_1_S_2_P_3_T_4_S_5_P_6_). However, despite the sequence variation, the antibody still recognized Rpb1-Ser5p in *Cn*, and SY-1365 treatment for 1 h reduced Rpb1-Ser5 phosphorylation by ~4-fold (Fig. 7A; Fig. S4A). SY-1365 treatment for 1 h also reduced Rpb1-Ser2 phosphorylation by ~3-fold (Fig. 7B Fig. S4B). The Rpb1 signal for Ser2 and Ser5 returned after 2 and 3 h of drug treatment, respectively, consistent with the use of a carefully titrated sublethal drug dose to ensure that healthy cells are being examined. Taken together, the results demonstrate that *Cn*CDK7 has a role in regulating transcription initiation and elongation.

### Transcriptomic analysis corroborates phosphoproteomics and reveals a role for *Cn*CDK7 in mRNA splicing

**Fig 8:**
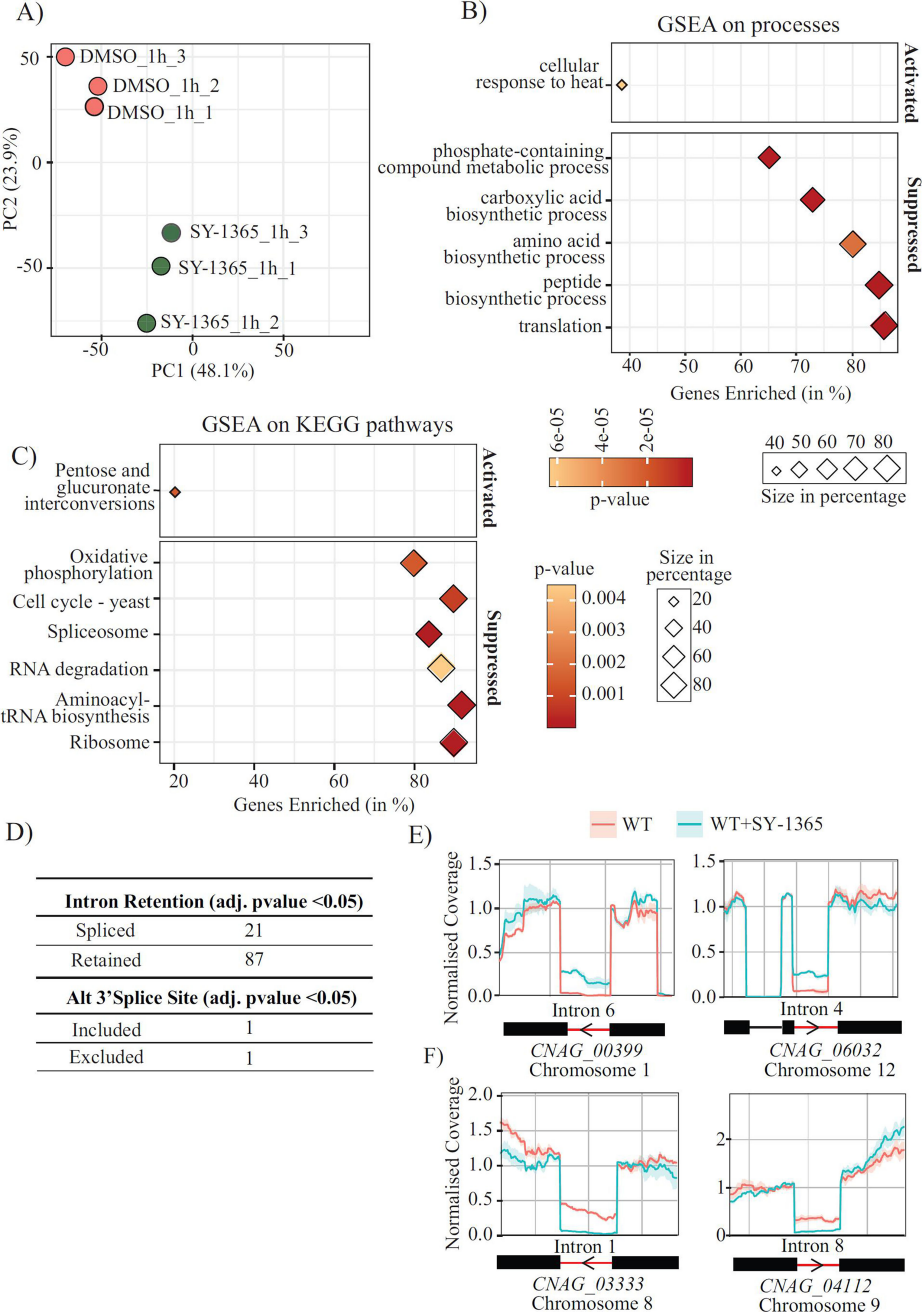
RNA-seq data analysis of SY-1365-treated *Cn* identifies CDK7 functions including splicing regulation. RNA-seq was performed on untreated and SY-1365-treated *Cn* (*n* = 3) as described in Materials and Methods. (**A**) A principal component analysis plot shows that replicates correlate well, with each treatment group having a distinct cluster profile. Gene set enrichment analysis (GSEA) was performed using the clusterProfiler R package to identify Gene Ontology categories (**B**) and Kyoto Encyclopedia of Genes and Genomes (KEGG) pathways (**C**) that were significantly enriched following SY-1365 treatment. Diamond plots display the enriched pathways, where size represents the percentage of enriched genes and color indicates the statistical significance of enrichment. (**D**) Table presenting alternative splicing events (ASEs) determined upon SY-1365 treatment. Inhibition of *Cn*CDK7 leads to more intron retention events over intron exclusion. Normalized coverage plots of representative ASE with (**E**) intron retention and (**F**) intron exclusion upon SY-1365 treatment.

Using RNA-seq as a second omics approach to investigate *Cn*CDK7 function, we evaluated the impact of SY-1365 on global transcription in *Cn* using RNA-seq (Fig. 8). A 1 h treatment time was chosen because it caused a marked reduction in Ser5 and Ser2 phosphorylation of RNAPII (Fig. 7). A principal component analysis plot showed good correlation within replicates, with each treatment having a distinct profile (Fig. 8A). Next, we performed gene set enrichment analysis (GSEA) to identify differentially regulated processes and pathways using the R package clusterProfiler (47). The genes used in the GSEA analysis are listed in Table S5. In agreement with the phosphoproteomics data, Gene Ontology enrichment analysis revealed that SY-1365 treatment suppressed fundamental processes, including translation and various energy-dependent biosynthetic pathways (Fig. 8B). SY-1365 also activated a small subset of genes involved in heat stress, suggesting that the drug elicits a general stress response (Fig. 8B).

Similarly, GSEA analysis of Kyoto Encyclopedia of Genes and Genomes pathways revealed that inhibiting CDK7 significantly downregulated translation-related processes, including ribosome biogenesis and tRNA synthesis. The cell cycle, which is known to be regulated by CDK7, was also downregulated by SY-1365, while the pentose and glucuronate interconversion pathway involved in biosynthetic processes and detoxification was activated (Fig. 8C). Additionally, pathways related to RNA splicing and also observed in the phosphoproteomics and RNA degradation, both essential co-transcriptional quality control mechanisms (48), were suppressed. This coordinated downregulation suggests that RNA homeostasis is disrupted following CDK7 inhibition.

To further investigate the role of *Cn*CDK7 in splicing, we reevaluated the RNA sequencing data to compare the frequency of alternative splicing events (ASEs) in drug-treated and untreated *Cn* using the R package SpliceWiz (49). ASEs were quantified using the percent spliced-in (PSI) metric. The analysis revealed a statistically significant increase in ASEs following drug treatment [abs(log_2_ fold change [log_2_FC]) >1, ΔPSI >5%, adjusted *P* < 0.05], with intron retention (IR) emerging as the predominant splicing event compared to other types, such as alternative 3′ splice site usage (Fig. 8D; Table S6). This is consistent with intron retention being the most induced ASE in *Cn* in response to environmental stress (50). Figure. 8E presents representative coverage plots of significant events, including intron retention (*CNAG_00399* and *CNAG_06032*) and intron exclusion (*CNAG_03333* and *CNAG_04112*). These findings highlight a regulatory role of *Cn*CDK7 in mRNA splicing.

### Inhibiting *Cn*CDK7 slows cell cycle progression through the G_2_/M phase

**Fig 9:**
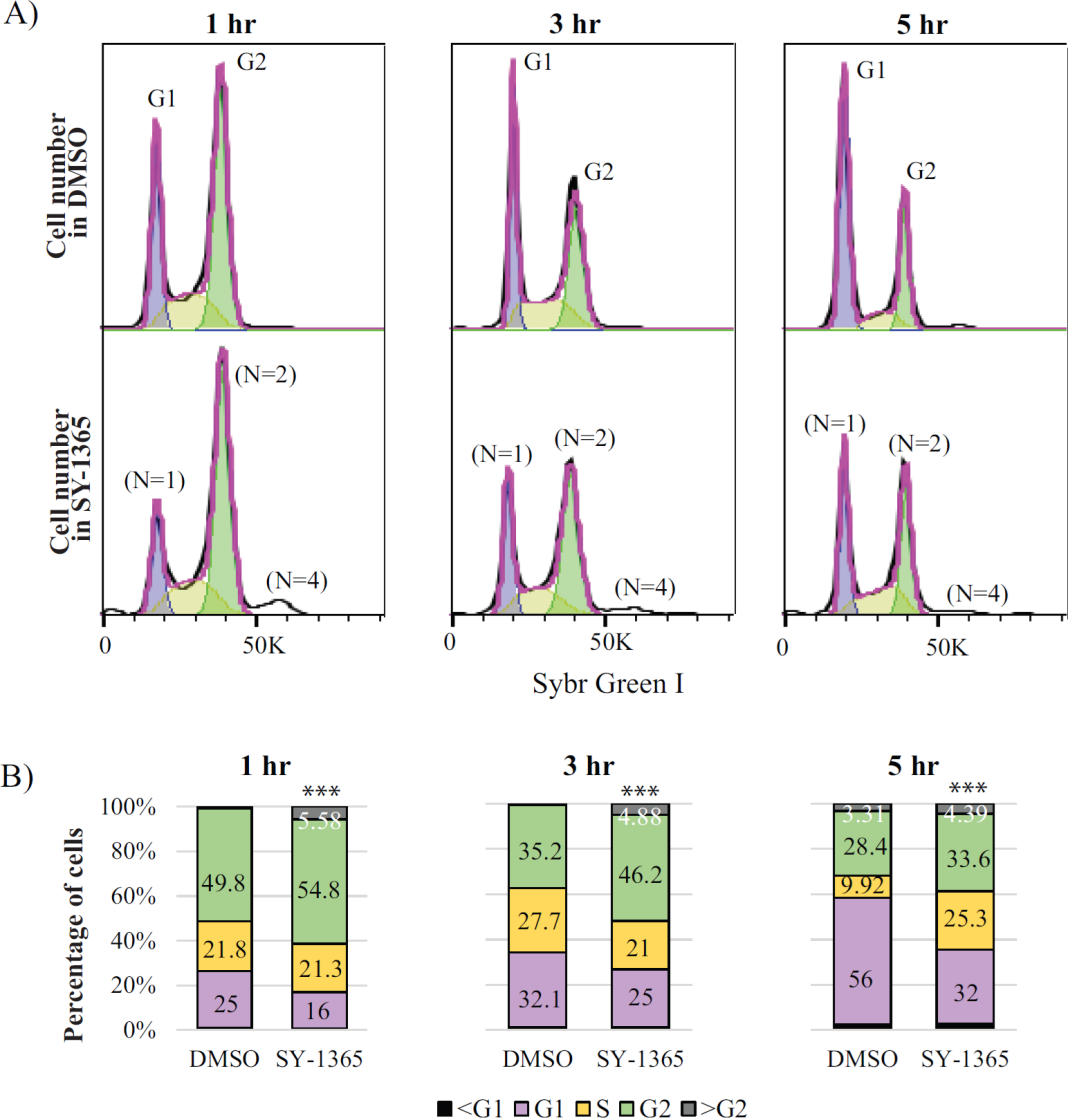
Effect of SY-1365 on the *Cn* H99 cell cycle. Cells in the log phase were fixed and stained with SYBR Green, and gated single cells (see Fig. S5) were analyzed by flow cytometry. (**A**) Histograms of duplicate samples showing a decrease and increase in the number of cells in G1 (*n* = 1) and G2 (*n* = 2), respectively, after 1, 3, and 5 h treatment with SY-1365 as compared to untreated cells (0.1% DMSO). Drug treatment also led to an accumulation of cells with a DNA content greater than G2 (*n* = 4). Purple- and green-shaded peaks depict the G1 and G2 phases, respectively, and the yellow-shaded curves depict the S phase. (**B**) Graphic representation of the percentage of *Cn* in each cell cycle phase, with the percentage indicated only if it is greater than 1%. Asterisks (***) indicate that the growth phase differences between SY-1365-treated *Cn* and untreated *Cn* (DMSO) are statistically significant (***P < 0.001, chi-square test).

Phosphoproteomics revealed reduced phosphorylation of the cell cycle regulators Cdc24 (51) and Cwf19 (42) following SY-1365 treatment (Fig. 6A; Table S4), while transcriptome analysis indicated overall suppression of the cell cycle following SY-1365 treatment (Fig. 8). To determine how the cell cycle is impacted by *Cn*CDK7 inhibition, *Cn* was treated with SY-1365 or DMSO for 1, 3, and 5 h, and the proportion of cells in each cell cycle phase was compared using flow cytometry (Fig. 9). Growth curves (Fig. S5A) and cell images (Fig. S5B) indicate that, prior to permeabilization and staining with SYBR Green for flow cytometric analysis, treated and untreated *Cn* were in log phase and did not take up DAPI. This is consistent with the presence of healthy cells with an intact cell wall and use of a sublethal dose of SY-1365. Untreated *Cn* had a doubling time in log phase of ~120 min, in agreement with what others have found for *Cn* (132 ± 16) (52), and drug-treated cells grew slower than untreated cells. After gating on the single-cell population and excluding budding cells, a plot of forward scatter vs SYBR Green fluorescence identified three distinct cell populations corresponding to *n* = 1, *n* = 2, and *n* = 4 (Fig. S5C). The histograms indicate that the percentage of these single cells that were in the G_1_ phase (*n* = 1) after 1 h of drug treatment was 16% compared to 25% for the DMSO-treated control. This coincided with an increase in the proportion of cells in the G_2_ phase (*n* = 2), from 50% to 55%, and with DNA content greater than G_2_ (*n* = 4) (Fig. 9A and B). After 3 and 5 h of treatment, the proportion of cells in G_1_ was reduced to 25% and 32%, from 32% and 56%, respectively, for the DMSO control, and the proportion of cells in G_2_ was increased to 46% and 34%, from 36% and 28%, respectively, for the DMSO control. The proportion of cells in the *n* = 4 population increased most significantly following 1 and 3 h of treatment with SY-1365. The microscopic images demonstrate that multi-budded cells are absent in drug-treated *Cn* (Fig. S5B). This is consistent with N2 and N4 cells being ploidy without having completed cytokinesis. Polyploidy is a common feature of *Cn* and has been reported to occur in fluconazole-treated *Cn* (53, 54). Interestingly, we did not observe that drug-treated cells were enlarged due to polyploidy.

**Fig 10:**
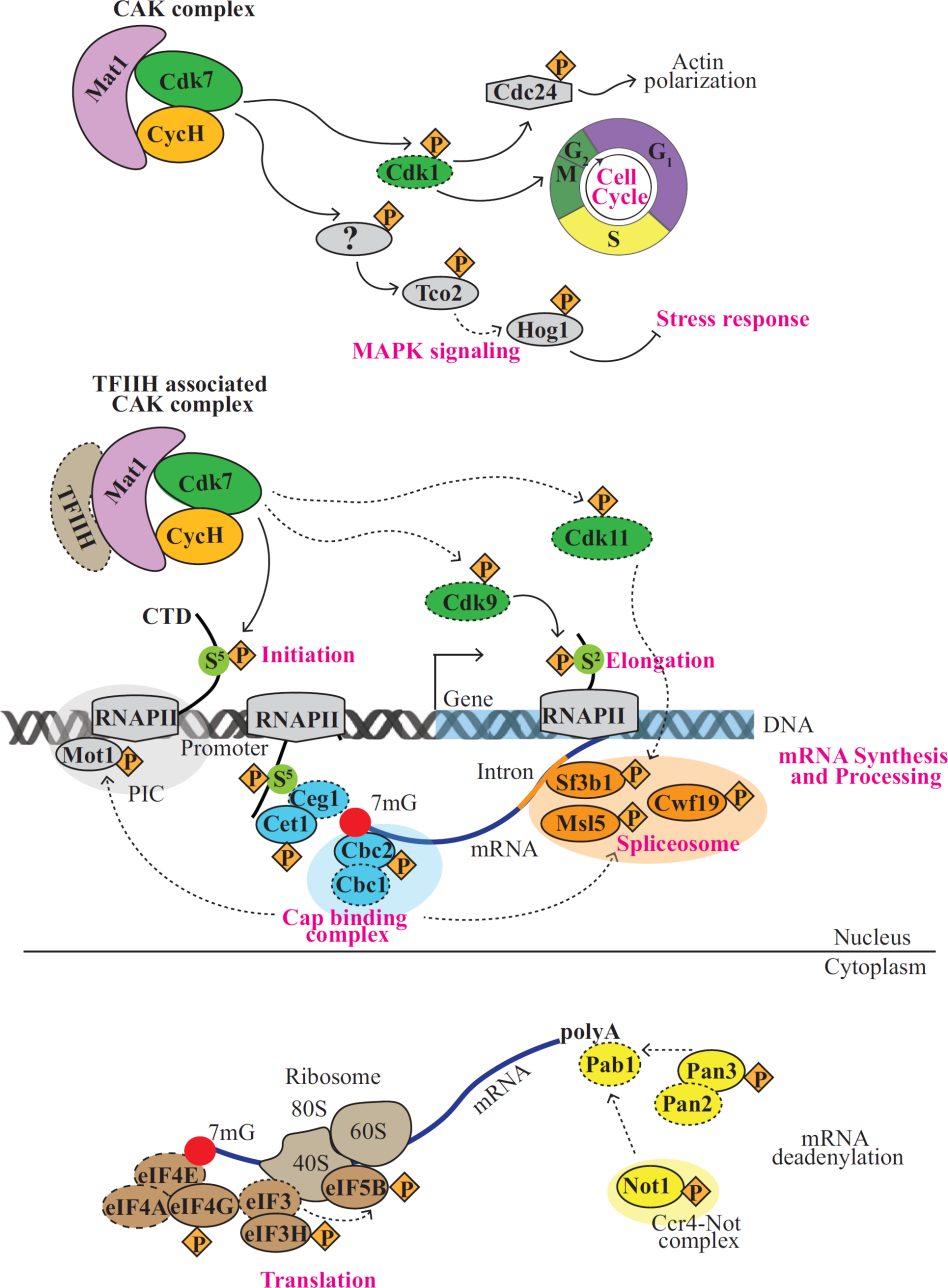
Model depicting the role of *Cn*CDK7 in regulating the cell cycle, transcriptional processes, and translation. Cell cycle regulation: in the nucleus, *Cn*CDK7 in the CAK complex activates Cdk1 to promote cell cycle progression through G_2_/M. Activated Cdk1 also phosphorylates Cdc24 to promote actin polymerization. *Cn*CDK7 either directly phosphorylates components of the Hog1 signaling pathway or acts through an intermediary protein affected by *Cn*CDK7-dependent phosphorylation. Transcription: also in the nucleus, TFIIH-associated CAK phosphorylates Ser5 on the CTD of the Rpb1 subunit of RNAPII (see Fig. 7), allowing RNAPII release from the promoter to initiate transcription. Ser5 phosphorylated-RNAPII pauses ~30 bp post-initiation and interacts with the capping enzymes, Ceg1p (RNA guanylyltransferase) and Cet1p (RNA triphosphatase), to form a capping complex. This complex allows the co-transcriptional formation of the 7-methylguanosine (7 mG) cap (red circle) on the 5′ end of newly synthesized (nascent) mRNA, ensuring mRNA stability, nuclear export, and translation. Cbc1 (mammalian Cbp80 homolog) and Cbc2 (mammalian Cbp20 homolog) then form a heterodimeric cap binding complex (CBC) that binds to the 7mG cap to stimulate formation of the pre-initiation complex (PIC) via the transcription regulator, Mot1. Post-release of the RNAPII transcriptional pause, *Cn*CDK7 is essential for Ser2 phosphorylation of Rpb1 to allow transcription elongation (see Fig. 7). This occurs via *Cn*CDK7-mediated activation of CDK9. The CBC also links capping to splicing by promoting the recruitment of U1 snRNP to the 5′ splice site to initiate spliceosome assembly. The spliceosome, comprising U1–U6 snRNPs, Sf3b1, Msl5 (binds to the branch point sequences of the intron), and Cwf19 (facilitates spliceosome disassembly and mRNA release), mediates intron removal. As in humans, *Cn*CDK7 may also enhance spliceosome maturation by activating *Cn*CDK11 to phosphorylate Sf3b1 (55). Translation: in the cytoplasm, Pab1 binds the 3′ poly(A) tail of mature mRNAs, protecting them from degradation. Two major deadenylation complexes then act sequentially to regulate mRNA stability and turnover: the Pan2/Pan3 complex trims the poly(A) tail, while the Ccr4-NOT complex trims it further, leading to mRNA decay. The CBC at the 5′ end is replaced by eIF4E, which, along with eIF4A and eIF4G, initiates recruitment of additional eIFs to assemble ribosomal subunits on the mRNA, ultimately leading to the formation of a functional ribosome and the initiation of translation. Proteins with solid lines were identified in the CDK7 phosphoproteome (see Fig. 6), while proteins, interactions, and functions delineated by broken lines are based on studies in higher eukaryotes and the presence of the homologous protein in *Cn*.

Taken together, SY-1365 impacts *Cn* growth by inhibiting *Cn*CDK7, which leads to G_2_/M arrest and activation of Hog1 signaling. The inhibition of *Cn*CDK7 with SY-1365 also prevents phosphorylation of the CTD of the Rpb1 subunit of RNAPII at both Ser5 and Ser2, suppressing transcription initiation and elongation, co-transcriptional processes including splicing, and translation, as summarized in the model in Fig. 10.

## DISCUSSION

This study confirmed that adding molecular tags to the *Cn* homologs of CDK7, cyclin H, and Mat1 does not impair fungal growth or the virulence composite and that these components assemble into a trimeric CAK complex that localizes to the nucleus. Furthermore, tagging does not compromise CAK function, as CDK7 inhibitors inhibit growth of both the tagged and untagged strains equally, and the immunoprecipitated CAK complex is enzymatically active.

To study CAK function for the first time in *Cn*, the tagged CAK complex was used to identify human CDK7 inhibitors that also inhibit *Cn*CDK7. Similar to findings in human studies (11), we did not find a correlation between the degree of suppression of relative *Cn*CDK7 activity by the inhibitors and the presence of a covalent warhead, which in THZ1 and SY-1365 attaches to a non-catalytic cysteine (Cys312) outside the ATP-binding pocket (11, 56). This is also demonstrated by the fact that SY-5609 (CDK7-IN-3), which lacks the covalent warhead (27, 28, 57), suppressed the relative activity of *Cn*CDK7 to a greater extent than SY-1365. Interestingly, *Cn*CDK7 lacks a potential recipient cysteine to enable covalent attachment, suggesting that inhibition of *Cn*CDK7 by SY-1365, CDK7-IN-1, and YKL-5-124 does not involve covalent attachment or that covalent attachment occurs via a less common target such as lysine or the N-terminal amino group (58).

Instead of implicating differences in antifungal activity to the presence or absence of the covalent warhead, our findings suggest that differences in antifungal activity between SY-1365, CDK7-IN-3, and samuraciclib likely stem from variable fungal cell penetration. This is because low concentrations of these inhibitors synergize with licensed antifungals, particularly AmB, despite each having a different baseline enzyme inhibitory and/or antifungal activity as a stand-alone agent. Our studies identified SY-1365 as a suitable, bioavailable inhibitor for studying CDK7 function in *Cn*. SY-1365 also potently kills tumor cell lines, particularly of leukemic origin (26), and our data now highlight the dual potential of CDK7 inhibitors as chemotherapeutic agents and antifungals, particularly in blood cancer patients at risk of opportunistic infections, including with *Cn* (59). Our assays also provide a foundation for developing even more potent and/or bioavailable inhibitors of *Cn*CDK7 as stand-alone antifungal agents.

SY-1365 was used to compare CDK7 function in *Cn* with previous findings in model yeast and humans. Specifically, Western blotting showed that inhibiting *Cn*CDK7 with SY-1365 reduced phosphorylation of Rpb1, consistent with the *in vitro* kinase assay results. Similarly, SY-1365 treatment of a human cancer cell line reduced Rpb1 phosphorylation without changing total Rpb1 levels (15). In that study, total Rpb1 remained unchanged over 24 h of drug exposure. Although we were unable to measure total Rpb1 in *Cn*, the human data are consistent with total Rpb1 in *Cn* also being unaffected by the shorter (0.5–1.0 h) treatment we used to capture the transient post-translational modification of Rpb1. Our Western blotting results are consistent with roles for *Cn*CDK7 in transcription initiation (promoter escape) and elongation in association with CDK9. The reduction in Ser2 phosphorylation implies that *Cn*CDK9 (CNAG_05549) is a substrate of *Cn*CDK7. This is similar to human CDK7, which phosphorylates the T-loop of CDK9 (9, 56) and contrasts with both *S. cerevisiae* and *S. pombe*, which use a separate CAK (*Sc*Cak1 and *Sp*Csk1) to activate CDKs involved in transcription, including their CDK9 orthologs (60, 61). The Western blotting results are supported by our omics data, where SY-1365 treatment suppressed transcription-associated functions. Furthermore, both omics approaches implicated CDK7 in promoting post-transcriptional processes such as RNA splicing and capping, as has been shown for Kin28 in *S. cerevisiae* (18), translation, and cell cycle regulation, with a splicing defect confirmed following analysis of the RNA-seq data. In *Cn*, splicing has been implicated in controlling gene expression, not proteome diversity (50). Hence, SY-1365-induced retention of introns would have a similar impact on gene expression and translation as preventing transcription initiation and elongation.

Phosphoproteomic analysis of SY-1365-treated *Cn* revealed the first evidence of a link between CDK7 function and Hog1 signaling. Bahn et al. proposed two hypotheses to explain stress-induced Hog1 activation via its dephosphorylation: (i) that it is initiated by phosphotyrosine or phosphoserine/threonine (Ptc) phosphatases activated by Hog1 itself or (ii) that Hog1 dephosphorylation occurs directly in response to stress (45, 46). We found that SY-1365 treatment suppresses the phosphorylation of both Tco2 and Ptc2. Repetto et al*.* demonstrated that a yeast CDK (Cdc28/CDK1) involved in cell cycle regulation synergizes with a pheromone-responsive MAPK (Fus3) to regulate Ste5 via phosphorylation within a shared multi-phosphorylation site region (62). However, whether CDK7 directly phosphorylates components of the Hog1 signaling pathway or acts through an intermediary protein affected by CDK7-dependent phosphorylation remains to be determined.

SY-1365 treatment caused an accumulation of *Cn* cells in the G_2_/M phase, a concomitant reduction of *Cn* cells in the G_1_ phase. This contrasts to *S. cerevisiae*, where Kin28 has no reported role in cell cycle regulation (15) and humans, where, in several breast cancer lines, SY-1365 treatment resulted in a different cell cycle profile characterized by cell accumulation not only in G_0_/G_1_ but also in G_2_/M due to a decline in the proportion of cells in the S phase (63). The differences in the cell cycle profile observed for *Cn* and human cells post-SY-1365 treatment could be due to all stages of the cell cycle being controlled by a single CDK (CDK1/CDC2) working in concert with different cyclins at each cell cycle phase (64–66). This contrasts with mammalian cells where the cell cycle is controlled by multiple CDKs, all of which are regulated by CDK7 phosphorylation.

Although human CDK7 inhibitors are potent and specific for human CDK7, they have been proposed to exhibit some off-target effects by inhibiting other human CDKs (11). From an antifungal treatment perspective, particularly in the context of treating opportunistic fungal infections in cancer patients, these off-target effects are unlikely to be of concern. However, they should be considered when using CDK7 inhibitors to study CDK7 function in all eukaryotes, including *Cn*, as they may confound interpretation even though there are only 6–8 fungal CDKs and 20 in human cells (4).

In summary, our study demonstrates that CDK7 function in *Cn* is more similar to that in humans than in model yeasts and may reflect the presence of introns in *Cn* genes, which are largely absent in model yeast. We also provide proof of concept that CDK7 inhibitors developed for human cancer treatment, including two that are orally administered, could be repurposed or dual-purposed as antifungal agents when combined with licensed antifungals. This potential is supported by the high sequence similarity between human and *Cn*CDK7 and by the observation that CDK7 inhibitors suppress both human and *Cn*CDK7 enzyme activity. The inhibitor, SY-1365, also serves as a valuable tool for determining the divergent functions of CDK7 in *Cn* compared to non-pathogenic yeast. Our findings suggest that the antifungal activity of SY-1365 likely results from cell cycle disruption and transcriptional impairment via interference with transcription initiation and elongation, splicing and capping, and mRNA stability and degradation, which ultimately impact translation.

## MATERIALS AND METHODS

### Strains, media, and inhibitors

The *C. neoformans* H99 strain was used for drug screening, antifungal growth and susceptibility testing, omics studies, Western blotting, and cell cycle analysis. The *Cn* KN99 strain, which was derived from H99 and is almost genetically identical with the same virulence profile *in vitro* and in animal models (67), was used to tag CDK7 and to demonstrate its subcellular localization. Construction and phenotypes of the *Cn*CDK7 triple-tagged strain in the KN99 background are described in the Supplemental Method, Table S1 through S3, Fig. S1. CDK7 inhibitors of ≥99% purity were purchased from MedChemExpress through Focus Bioscience Pty Ltd, Queensland, Australia. Where available, the salt forms of the inhibitors were purchased instead of the free forms as they are typically more soluble and stable. Stock solutions were prepared in 100% DMSO.

### Fluorescence microscopy

WT (KN99) and the CDK7 triple-tagged strains were grown overnight in yeast extract peptone dextrose (YPD) media. Cells were washed twice with sterile water, and the OD_600_ of the final suspension was measured. Cells were diluted to an OD_600_ of 1 in 3 mL minimal media (15 mM glucose, 10 mM MgSO_4_, 13 mM glycine, and 3 µM thiamine) with 29.4 mM KH_2_PO_4_, cultured for 3 h, and 500 µL was treated with DAPI (2 µL of 100 ng/mL) for 5 min. Excess media was removed by centrifugation, leaving some residual behind to form a thick slurry, and 7 µL was pipetted onto a microscope slide. Cells were viewed under the Delta Vision microscope at ×100 magnification using the appropriate channels for mNG and DAPI.

### Pulling down the CAK complex with NanoTrap beads

Pull-downs were performed as described (68). Briefly, the KN99 and the CDK7 triple-tagged strains were grown overnight in YPD and used to inoculate fresh YPD to an OD_600_ = 0.02. Cultures were incubated overnight (19–21 h), and an OD_600_ of 200 was pelleted and snap-frozen in liquid nitrogen. Pellet weight was recorded, and cells were resuspended in 1.5 volume of lysis buffer (0.1% NP-40, 250 mM NaCl, 5 mM EDTA, 50 mM Tris-HCl, pH 7.5, 1 mM dithiothreitol [DTT], and 50 mM NaF) containing 1 µL of protease inhibitor cocktail (Sigma, P8215) per 20 mg cell pellet. Lysates were prepared by bead-beating cells with 425–600 µm glass beads and removing debris by centrifuging at 16,000 × *g* at 4°C. ChromoTek mNeonGreen-Trap Agarose beads (cat. no. nta) and V5-Trap agarose beads (cat. no. v5ta) were used to pull down mNeonGreen-tagged CDK7 from the cleared lysates, with KN99 serving as a control for untagged CDK7. The mNeonGreen-Trap beads were pre-washed twice with lysis buffer and incubated with cleared lysates for ~2 h at 4°C. Following pull-down, beads were washed three times with lysis buffer. Pulled-down CAK complex was used for Western blotting and *in vitro* kinase assays.

### *In vitro* kinase assay using the pulled-down CAK complex

The pulled-down CAK complex was washed twice with kinase buffer (40 mM Tris, pH 7.5, 20 mM MgCl_2_, 100 µg/mL bovine serum albumin, and 2 mM DTT) for use in the assays. The reactions were performed at room temperature in kinase buffer containing 10 µM ATP and 250 µM of the peptide substrate, CDK7/9tide. Inhibitors were added at various concentrations in a final DMSO concentration of 5%. A reaction mastermix (kinase buffer, ATP, and 250 µM CDK7/9tide, excluding inhibitors) was prepared and added to the bead-bound CAK complex to start the reaction. At the indicated times, the reaction mixtures were centrifuged to pellet the beads, and 25 µL aliquots of the supernatant were mixed with 25 µL of Kinase-Glo reagent to stop the reaction and measure the remaining ATP as a relative luminescence unit (RLU). Luminescence was measured using a SpectraMax iD5 instrument with an integration time of 0.5 s. The RLU values were used to calculate the relative activity (%) of CDK7. Firstly, raw RLU values at each time point were normalized to the RLU at time 0 (baseline), which represents 100% ATP remaining (no ATP consumption). For each subsequent time point, the percentage of ATP remaining was calculated using the following formula:


$$ATP\;remaining\;\left(\%\right)=\left(\frac{RLU_{time}}{RLU_{time\;0}}\right)\times 100$$

Secondly, the percentage of ATP consumed was calculated by subtracting the ATP remaining from 100%:


ATP consumed (%)=100−ATP remaining(%)

Finally, to determine relative enzymatic activity in the presence of inhibitors, values were normalized to the “no drug” control sample, which is defined as 100% activity (no inhibition). Relative activity (%) for each treatment with inhibitor was calculated as:


$$Relative\;activity\left(\%\right)=\left(\frac{ATP\;consumed_{Inhibitor}}{ATP\;consumed_{No\;drug}}\right)\times 100$$

### Screening for CDK7 inhibitors with antifungal properties

#### Broth culture

Strain H99 was grown overnight in YPD media at 30°C with shaking (250 rpm) and seeded in fresh YPD at an OD_600_ of 0.01. CDK7 inhibitors were added, and cells were incubated at 30°C with shaking. The final DMSO concentration was 0.5%. OD_600_ was measured at 17 h.

#### Dose–time–response assay in a 96-well plate

H99, KN99, and the triple-tagged strain were grown overnight in 5 mL YPD media at 30°C. The cells were diluted in fresh YPD to an OD_600_ of 0.01 and aliquoted into tubes. Serial twofold dilutions of the compound SY-1365 were added to each tube, and one tube received vehicle only (0.5% DMSO). Two hundred microliters of drug-treated and untreated cells were aliquoted into a flat-bottom 96-well plate. Cell growth under constant shaking (800 rpm) at 30°C was monitored by recording the OD_600_ every 20 min using an Agilent Biotek log phase 600 plate reader for 28 h. An R package, growkar (https://sethiyap.github.io/growkar/), was implemented to plot cell growth as time vs OD_600_.

### Antifungal susceptibility testing

Antifungal susceptibility testing was assessed using the European Committee on Antimicrobial Susceptibility Testing method. Growth was assessed after 3 days at 37°C. The minimum inhibitory concentration (MIC) is defined as the lowest drug concentration to inhibit growth by 100%. The FICI is the antifungal effect of combination therapy.

### Liquid chromatography–mass spectrometry and phosphoproteomics

#### Sample preparation

An overnight culture of H99 was pelleted by centrifugation. The pellet was washed twice with water and resuspended at OD_600_ = 3/mL in 20 mL YPD, and the cells were grown for 2 h at 30°C. SY-1365 (60 µg/mL) was added to the treatment samples, and all cultures were incubated for a further 5 h at 30°C. Cells of OD_600_ = 100 were harvested in duplicate and snap-frozen in liquid nitrogen. Pellets were resuspended in sodium deoxycholate (SDC) lysis buffer (4% SDC, 100 mM Tris-HCl, pH 8.5, phosSTOP) and lysed by bead-beating. Samples were centrifuged at 16,000 × *g* for 10 min, and the supernatants were collected. A subset of each sample underwent protein precipitation and bicinchoninic acid assay for protein estimation. Samples were submitted to the Sydney Mass Spectrometry Facility, with half of the sample used for 1D-liquid chromatography–mass spectrometry (LCMS) and the other half for phosphoproteomics.

#### LCMS and Phosphoproteomics

Phosphoenrichment was performed as described (69) (see the Supplemental Method). LCMS and phosphoproteomics data analysis are explained in the Supplemental Method and Fig. S4.

### Western blotting to detect phosphorylated Rpb1 and Hog1

H99 was grown overnight in YPD media. An OD_600_ = 4/mL of cells was resuspended in fresh YPD and grown for 2 h at 30°C with shaking. Cells were treated with 60 µg/mL of SY-1365. An OD_600_ = 20 of cells was harvested at each time point, snap-frozen in liquid N_2_, and stored at −80°C. Protein was extracted using TRIzol as described (70). TRIzol-extracted protein was subjected to SDS-PAGE (70, 71). Ser5p Rpb1 was detected by incubating the blot overnight with monoclonal antibody against phosphor-S5 of the Rpb1 subunit of RNAPII (3E8-ab252852; 1:1,000 dilution in tris-buffered saline with Tween 20 [TBST]), followed by horseradish peroxidase (HRP)-linked anti-rat Ab (ab6734; 1:7,500 dilution in TBST) for 1 h. Histone was detected by antihistone H3 Ab (ab1791) followed by HRP-linked antirabbit Ab (NA9340V; 1:7,500 dilution in TBST) as a loading control. Total and phospho-Hog1 were detected by anti-Hog1 (y-215; sc-9079; 1:1,000) and antiphospho-p38 MAPK Ab (Thr180/Tyr182) (D3F9), respectively, followed by HRP-linked antirabbit Ab (cat. no. NA9340V; 1:7,500 dilution). Ser2p Rpb1 was detected by Ser2p (Pol2RA)30888-1-AP followed by HRP-antirabbit Ab (NA9340V; 1:7,500). Cdc2 was detected by anti-Cdk1/Cdc2 (PSTAIR) (06-923, 1:400) followed by HRP-antirabbit Ab (NA9340V; 1:7,500). Chemiluminescence was detected using a ChemiDoc imaging system (Bio-Rad) following membrane incubation with Amersham ECL Western Blotting Detection Reagents (Cytiva RPN2106).

### RNA extraction and library preparation

H99 was grown in YPD medium overnight at 30°C with shaking (250 rpm). An OD_600_ = 4/mL of cells was resuspended in triplicate in fresh YPD and grown for 2 h, and then treated with SY-1365 (60 µg/mL) for 1 h. Cell pellets were harvested, snap-frozen in liquid nitrogen, and stored at −80°C. RNA was extracted using TRIzol reagent (72), purified using a Qiagen RNeasy Mini-Elute Cleanup Kit (cat. no. 74104), and quantified using Agile.”nt TapeStation RNA screen tape to ensure sufficient RNA integrity for library preparation. RNA (1 µg) with a RNA integrity number (RIN) score of >8 was used to prepare poly-A enriched RNA libraries following the manufacturer’s protocol for the Illumina stranded mRNA prep kit (cat. no. 20040534). Libraries for different samples were barcoded with Illumina index primers provided in Illumina RNA UD Indexes Set A. The quality of each library based on size, distribution, and yield was assessed using Agilent TapeStation DNA 1000 screen tape. RNA sequencing libraries were pooled in equimolar ratios and sequenced on Illumina Novaseq X 300 (150 bp paired end) platform at AGRF.

### RNA sequencing analysis

RNA-seq data analysis was performed as previously described (72, 73). Briefly, quality of raw fastq reads (deposited to NCBI SRA database, accession number PRJNA1293871) was assessed by FastQC (74), and adaptors were trimmed using a fastQ pre-processor tool: fastp (v.0.19.6) (75). Trimmed fastq reads were aligned to the *C. neoformans* genome (v.FungiDB-59_CneoformansH99) using the STAR (v.2.7.8 a) splice read aligner (76). Raw read counts for each gene in all the samples and replicates were obtained by an R package Rsubread (77). Differential gene expression analysis on normalized read counts was performed using DESeq2 (v.1.42.1) (78) implemented in the RNA-seq data analysis R package parcutils (https://github.com/cparsania/parcutils) for SY-1365-treated samples over control WT samples. Genes with read counts of <10 were filtered prior to differential expression analysis, and log_2_ fold change was calculated for SY-1365-treated vs untreated samples. A custom *C. neoformans* H99 OrgDb object was created using the makeOrgPackage function from the AnnotationForge R package. GSEA was conducted using gseGO and gseKEGG functions in ClusterProfiler (*P* value cutoff: 0.05) (47), with the OrgDb object as background. Exploratory analysis was performed via the FungiExpresZ portal (79), and visualizations were generated using in-house R scripts (https://github.com/sethiyap/CnCDK7_analysis).

### Splicing analysis on RNA-seq data

Raw reads were mapped to the *Cn* reference genome (Cryptococcus_neoformans_var_grubii_h99_gca_000149245.CNA3.dna.genome.fa) obtained from the Ensembl database using STAR aligner (v.2.7.8 a) (76). ASEs were determined using an R package, SpliceWiz (49). BAM files of mapped reads were processed using the processBAM function in SpliceWiz. ASE events, including IR, were quantified using PSI values, calculated as follows:


$$PSI=\frac{Included\;event}{Included\;event+excluded\;event}$$

To determine differentially spliced events, ∆PSI was calculated as follows:


ΔPSI=PSI(treatment)−PSI(control)

The generalized linear model-based statistical tool, EdgeR (80), implemented in SpliceWiz, was used to identify differential ASE and log_2_FC values. Events with log_2_FC of >1 and log_2_FC of <−1 were referred to as “inclusion” and “exclusion” of ASE, respectively. An ASE was considered significant if the absolute ∆PSI was ≥5% (50); i.e., the difference in PSI values between treatment and control should be at least 5%, and the false discovery rate (FDR) should be <0.05 (49). Each significant splicing event was further categorized into specific ASE types based on the SpliceWiz output. Differential splicing between treated and untreated samples was visualized using coverage plots generated by the GetCoverageData function from the SpliceWiz package. Scripts for splicing analysis and visualization are available at https://github.com/sethiyap/SpliceFungi .

### Flow cytometry and cell cycle analysis

A H99 overnight culture was pelleted, washed twice in water, resuspended in 3 mL of YPD at OD_600_ = 3, and incubated for 2 h at 30°C with shaking before adding 30 µg/mL SY-1365 or 0.1% DMSO (control) and incubating for a further 1, 3, or 5 h. For growth curves confirming that cells are in log phase (*n* = 2 biological replicates) and cell morphological analysis using fluorescence microscopy, see Fig. S5. For fixation, cell pellets were washed in water and fixed in 75% ethanol for at least 24 h. Cell pellets were washed twice in 50 mM Tris-HCl, pH 7.5, then incubated for 150 min at 37°C with shaking (150 rpm) in 0.5 mg/mL RNase A (freshly boiled), 1× SYBR Green I, 0.25 M sucrose, 1 mM EDTA, 1 mM CaCl_2_, 2.12 mM MgCl_2_, 28 mM Tris-HCl, pH 7.5 (modified normal saline buffer from reference 81). Pellets were washed twice and resuspended in 1 mL of 50 mM Tris-HCl, pH 7.5. Samples were sonicated for 5 min in an ultrasonic tank. Duplicate samples were run on the BD FACS Canto II System, and gated single cells (see Fig. S5) were analyzed using the FlowJo cell cycle analysis function.

## Data Availability

Raw RNA-seq FASTQ files have been deposited in the National Center for Biotechnology Information Sequence Read Archive under BioProject accession number PRJNA1293871. All scripts used for processing and visualization of the RNA-seq, phosphoproteomics, flow cytometry, and growth curve data are available on GitHub (https://github.com/sethiyap/CnCDK7_analysis/blob/main/Paper_SY-1365.md). All data used to generate the figures are provided in the supplemental tables.

## SUPPLEMENTAL MATERIAL

Supplemental material mbio.02898-25-s0001.pdf Supplemental text and figures.

Supplemental Table mbio.02898-25-s0002.xlsx Tables S1 to S6.

ASM does not own the copyrights to Supplemental Material that may be linked to, or accessed through, an article. The authors have granted ASM a non-exclusive, world-wide license to publish the Supplemental Material files. Please contact the corresponding author directly for reuse.
