# Supplementary material for: Functional insight into cyclin-dependent kinase (CDK)7 via chemical inhibition of the priority fungal pathogen Cryptococcus neoformans
Source: mBio. 2025 Oct 31;16(12):e02898-25. doi: 10.1128/mbio.02898-25 (PMC12691695; doi:10.1128/mbio.02898-25)
Supplement: Supplemental material — Supplemental text and figures. [file mbio.02898-25-s0001.pdf]

Supplemental Method

Construction of the CDK7-tagged strain.

Molecular cloning was performed with HiFi DNA Assembly (NEB E2521L). Primers, plasmids and their construction details are listed in **Table S1** and **S2**. C. neoformans strains in KN99 background were constructed using the CRISPR/Cas9 system as described elsewhere [1]. DNA constructs for C-terminal tagging of CDK7 (CNAG_06445), Mat1 (CNAG_05877), and Cyclin H (CNAG_04405) were PCR amplified from the plasmids pBHM2406, pBHM2506, and pBHM2504, respectively, with primers MJB931 and MJB932, MJB961 and MJB944, or MJB962 and MJB938, respectively. All tagging constructs included 50 bp homology arms. sgRNAs with unique 20 bp spacers were constructed by amplifying U6 promoter and scaffold fragments individually from pBHM2329. U6-spacer fragments were amplified with the common forward primer MJB537 paired with either MJB930, MJB942, or MJB936. Spacer-scaffold fragments were amplified with the common reverse primer MJB538 paired with either MJB929, MJB941, or MJB935. Equal volumes of U6-spacer and spacer-scaffold fragments were then mixed and joined by fusion PCR with MJB535 and MJB536. The CnoCAS9 expression cassette was amplified from pBHM2403 with MJB537 and MJB538. All PCR products were purified using Nucleospin Gel and PCR Clean-up kits and eluted in water.

For transformation, *Cn* cultures were grown overnight to saturation in YPAD and were subcultured overnight in 100 mL of YPAD to reach an OD_{600} of 0.8-1.0 the following day, at which point they were harvested by centrifugation at 4°C. Pellets were then washed twice with ice cold water, followed by incubation on ice for 1 hr in 10 mL of ice cold electroporation buffer (10 mM Tris-HCl pH 7.5, 1 mM MgCl2, and 270 mM sucrose) and 1 mM DTT. After incubation, cells were pelleted by centrifugation and resuspended in 250 µL of ice-cold electroporation buffer. For each transformation, 45 µL of cell suspension was mixed with 2 µg of donor DNA, 700 ng of sgRNA, and 1 µg of CnoCAS9, and added to a pre-cooled 2 mm gap electroporation cuvette. Electroporation was performed at 500 V, 400 Ω, and 250 µF on a BTX Gemini X2 electroporator. Electroporated cells were resuspended in 1 mL of YPAD, incubated for 2 hr with rotation at 30°C, and then plated on YPAD containing 125 µg/mL nourseothricin sulfate (RPI N51200 or Jena Biosciences AB0-102XL), 300 µg/mL Hygromycin B (Calbiochem 400050 or Goldbio H-270-10), or 200 µg/mL G418 sulfate (Corning 61-234-RG). The first strain made was CDK7-mNG-CBP-2xFLAG-HYG (CM2444) which was used as the parent strain for the MAT1-6xHis-NEO (CM2446) transformation. CM2446 was then used as the parent strain for the CyclinH-V5-NAT (CM2448) transformation. Successful modifications were verified by diagnostic PCR.

References

1. Huang, M.Y., et al., Short homology-directed repair using optimized Cas9 in the pathogen Cryptococcus neoformans enables rapid gene deletion and tagging. Genetics, 2022. 220(1).

Figure S1

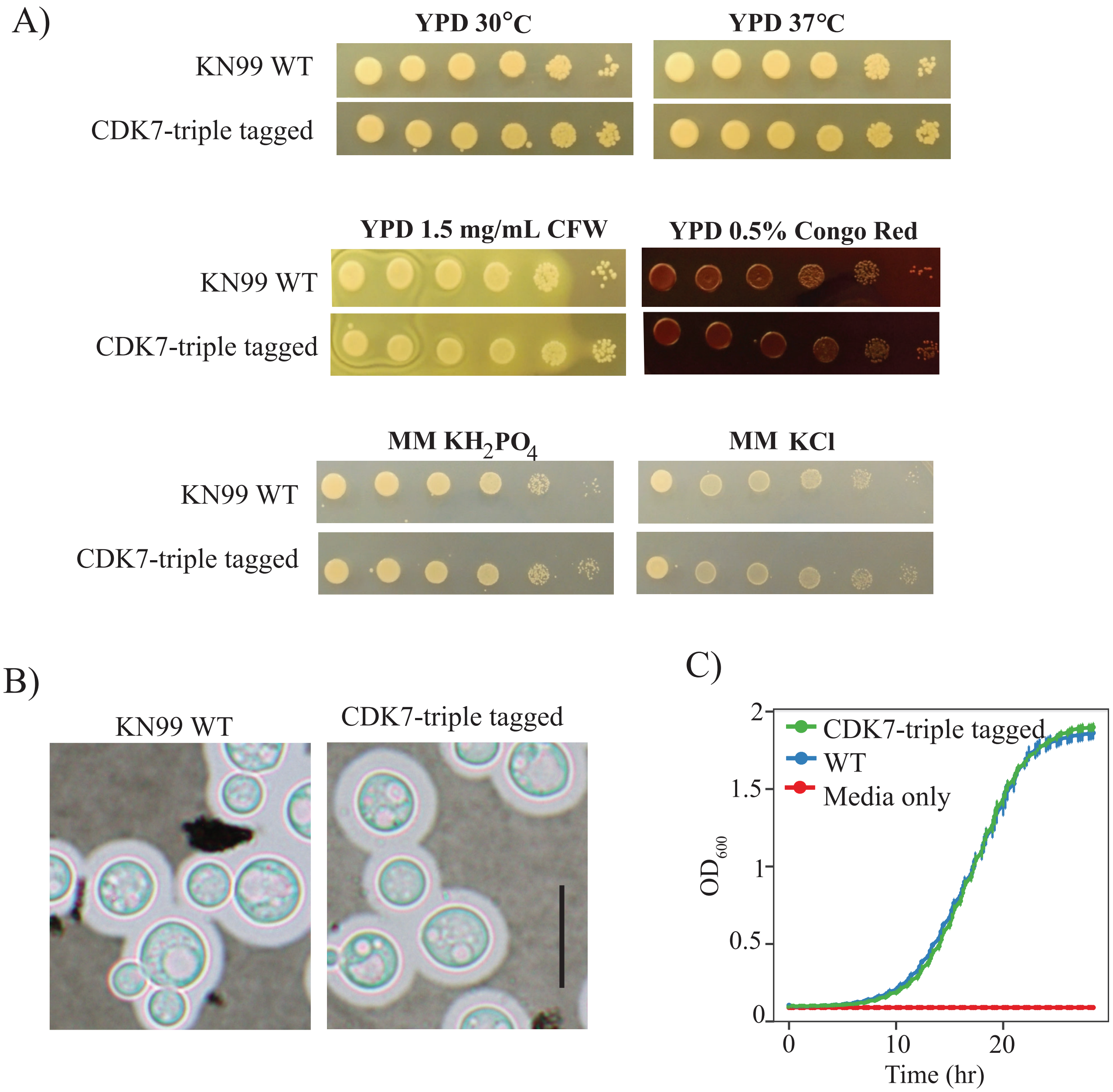

Figure S1. Tagging the CAK complex has no impact on *Cn* phenotypes. (A) Spot dilution assays confirm that the CDK7 triple-tagged strain grows at a similar rate to the parent WT strain KN99 under the conditions indicated. Cells were cultured overnight in YPD and adjusted to 10^6 cells per 3 µL (the amount used to spot). The cultures were subjected to 10-fold serial dilution, resulting in cell concentrations of 10^1 to 10^6 per 3 µL. The dilutions were spotted onto the agar medium indicated and incubated at 30°C or 37°C for 48–72 h. Calcofluor White (CFW) and Congo red are cell wall stressing agents. Minimal media (MM) KCl is phosphate-depleted as compared to MM with phosphate (MM KH_2PO_4). **(B)** KN99 and the triple-tagged strain have a similar cell and capsule morphology. Capsule was induced by overnight growth in MM KH_2PO_4 at 30°C with shaking (250 rpm). The cells were visualized under a light microscope after negative staining with India Ink. Scale bar = 10 µm. **(C)** Growth curves for the WT KN99 and CDK7 triple-tagged strains. Growth took place in a 96-well plate under constant shaking (800 rpm) at 30°C and was determined by recording OD_{600} readings every 20 min for 28 h using an Agilent Biotek log phase 600 plate reader.

Figure S2

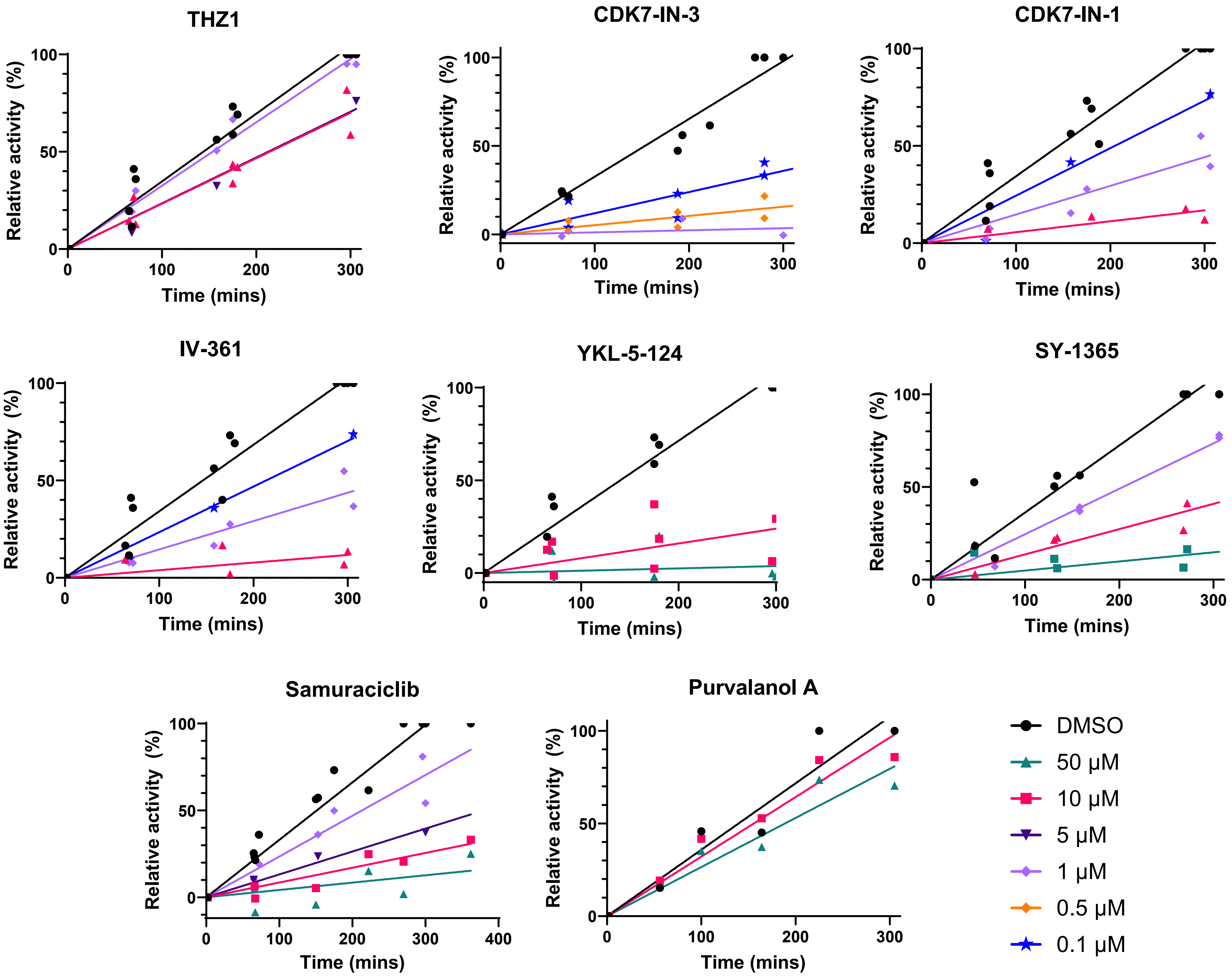

Figure S2. *Cn*CDK7 is inhibited by human CDK7 inhibitors. Kinase assays were performed using the concentrations of CDK7 inhibitor indicated, over 300 min, as described in Figure 4. CnCDK7 enzyme activity is expressed as **"Relative Activity (%)"** after normalization as described in the methods. n=2-3 independent experiments. The 300 min time point was used to generate Figure 4 with statistics.

Supplemental Method

Phosphoproteomics method (Figure S3).

Proteome and phosphoproteome data were processed using Proteome Discoverer 2.5, using the Label Free Quantitation feature, and MASCOT 2.8. The data was searched against the Cneoformans_H99 database supplied and an in-house "contaminants" database. Trypsin was selected as the enzyme, 30ppm as the mass tolerance and missed cleavage rate was set to 3. The fixed modifications were set to acetyl (N-term), carbamidomethyl (C), deamidated (NQ), oxidation (M), and phospho (ST) and phospho (Y) for the phosphoproteome data.

A total of 960 phospho-proteins belonging to *C. neoformans* were reported in the SILAC results. The dataset was filtered to retain only those with corresponding protein abundance data (904/960), and with peaks detected in both biological replicates (843/904). These 843 phospho-proteins were used for downstream analysis. Differential protein abundance for each phosphor-protein was determined by calculating Log_2 fold-change, Log_2FC (WT+SY-1365 vs WT) and proteins with $Log_2FC > 1$ or only detected in SY-1365 were denoted as "Up" and Log2FC < -1 or only detected in Untreated sample were denoted as "Down". Number of proteins with Up, Down and No-change in abundance are shown in **Figure S3A**. Given that the altered abundance of a specific phosphoprotein could be impacted by altered gene expression, 1D LCMS was performed in parallel on the same samples to determine protein abundance irrespective of phosphorylation status (**Figure S3B**). A total of 3,247 protein groups were identified. This dataset was filtered in two steps: (1) proteins with detectable peaks in at least two of the three replicates (n = 3,607), and (2) proteins with reported abundance values (n = 2,889). Log_2FC (WT + SY-1365 vs WT) values were determined to obtain differential protein abundance. Among these, ~90% (2,601 proteins) showed no significant change following drug treatment, while 102 were upregulated and 184 were downregulated (**Figure S3B**). To focus on proteins with altered phosphorylation but stable overall abundance after CDK7 inhibition, a Venn diagram analysis (**Figure S3C**) was performed using the 2,601 unchanged proteins (Figure S3B) and phosphoproteins identified across the three categories (**Figure S3A**). Down-regulated phospho-proteins with no-change in total protein levels (122) were referred to as the CDK7 phospho-proteome (**Table S4**), highlighted in red (**Figure S3C**).

Figure S3

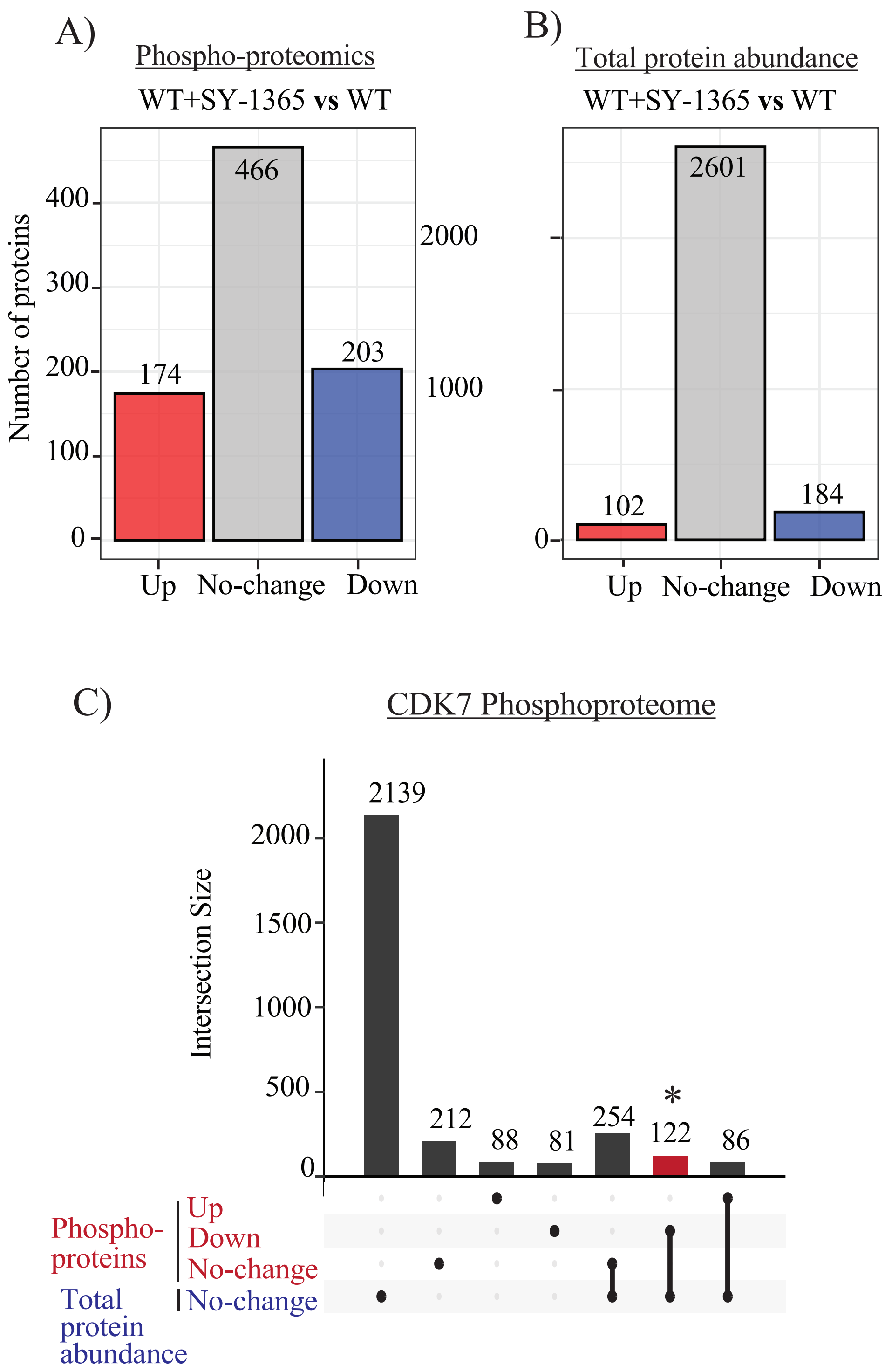

Figure S3: Phosphoproteomics analysis of SY-1365-treated Cn. (A) Bar plot showing changes in phosphorylation across the *Cn* phosphoproteome following SY-1365 treatment. Proteins in the "Up" category were hyper-phosphorylated by SY-1365 treatment or only detected in the SY-1365-treated sample ($Log_2FC > 1$). Those in the "Down" category were hypo-phosphorylated by treatment or only detected in the untreated sample ($Log_2FC < -1$). **(B)** Bar plot of proteins based on their differential abundance obtained by 1D LCMS. **(C)** Venn diagram showing overlap between proteins with unchanged total abundance (from **B**) and proteins with altered phosphorylation status (from **A**). Only proteins in the "Down" category with no change in protein abundance (red) were used for STRING analysis (**Figure 6A**).

Figure S4

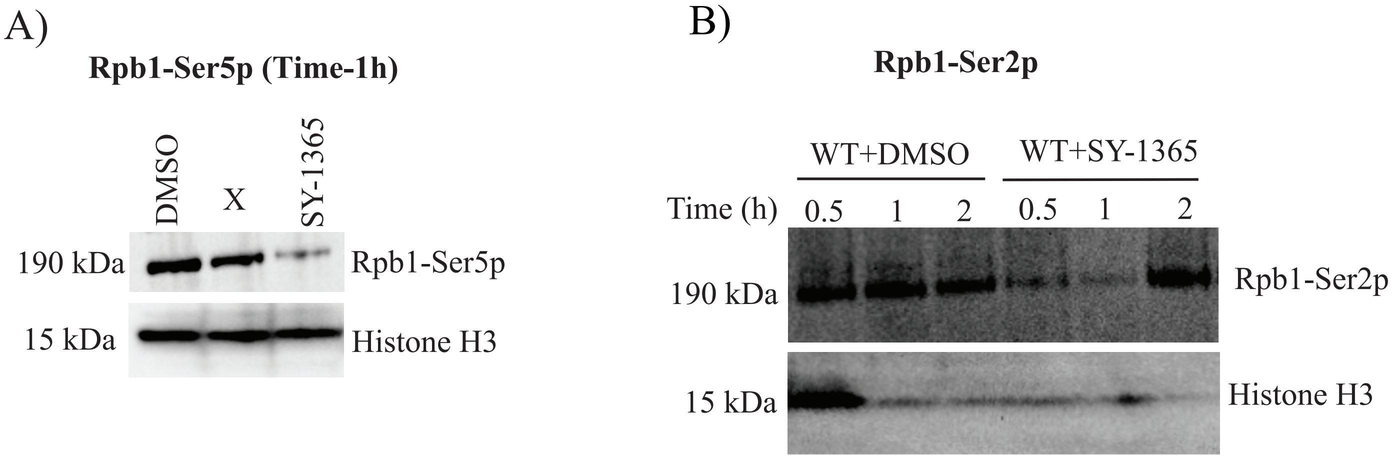

Figure S4: Replicate Western blot demonstrating that *Cn*CDK7 phosphorylates Ser5 and Ser2 in Rpb1. SY-1365 treatment inhibits phosphorylation of Rpb1 on Ser5 (Rpb1-Ser5p) within 1 h **(A)** and phosphorylation of Rpb1 on Ser2 (Rpb1-Ser2p) within 0.5 h **(B)**. In both **(A)** and **(B)**, the blots were re-probed with anti-Histone H3 antibody to detect Histone H3 (loading control)

Figure S5

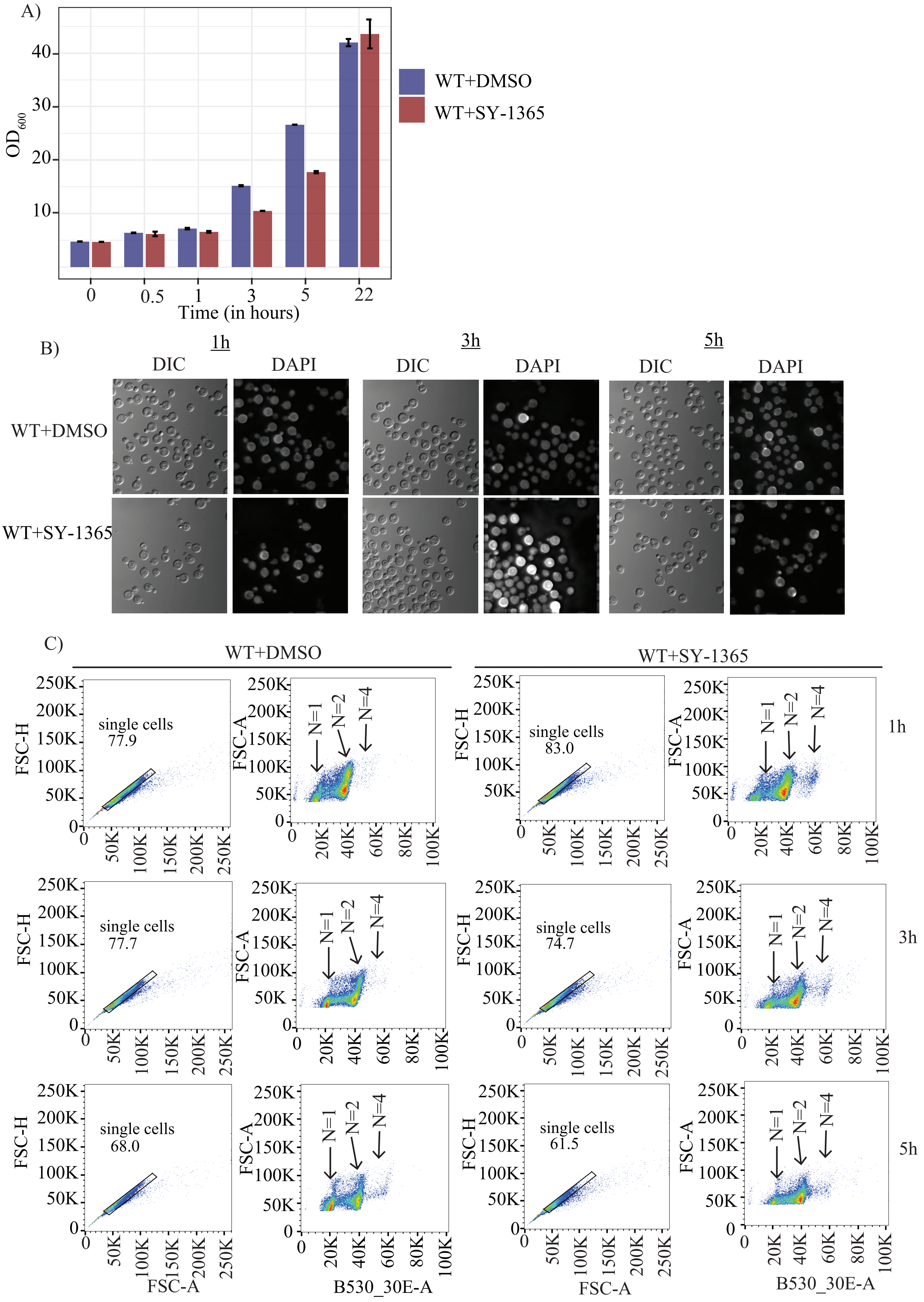
A)
OD600
Time (in hours)
0
0.5
1
3
5
22
10
20
30
40
WT+DMSO
WT+SY-1365
B)
1h
3h
5h
DIC
DAPI
WT+DMSO
WT+SY-1365
C)
WT+DMSO
WT+SY-1365
FSC-H
FSC-A
B530_30E-A
single cells
77.9
83.0
77.7
74.7
68.0
61.5
N=1
N=2
N=4
1h
3h
5h
0
50K
100K
150K
200K
250K
20K
40K
60K
80K

Figure S5: Analysis of untreated and SY-1365 treated *Cn* to establish that cells are in log phase and to demonstrate the gating strategy used, prior to flow cytometric analysis. (A) SY-1365-treated (30 µg/mL) and untreated cells were grown for 22h in biological duplicates, with growth assessed spectrophotometrically (OD_{600}) at the indicated time points. **(B)** Prior to fixation and staining *Cn* for flow, *Cn* morphology at 1h, 3h and 5h was assessed by fluorescence microscopy. In both treated and untreated samples, budding cells are observed (which were excluded from the flow analysis) and DAPI staining is confined to the cell periphery, confirming that cells are healthy and intact. **(C)** A plot of forward scatter height (FSC-H) versus forward scatter area (FSC-A) (see panels on left hand side) and gating on single (non-budding) cells for cell cycle analysis using flow cytometry. Right hand panels show a plot of the fluorescence intensity of the gated single cells [SYBR-I channel (B530_30E-A) versus FSC-A] where 3 distinct cell populations are observed (N=1, N=2 and N=4), with the proportion of N=4 cells being higher in drug-treated cells.
